# Comparison of artificial intelligence models and physicians in patient education for varicocele embolization: a double-blind randomized controlled trial

**DOI:** 10.3389/fradi.2025.1682725

**Published:** 2025-10-14

**Authors:** Ozgur Genc, Omer Naci Tabakci

**Affiliations:** ^1^ Department of Radiology, Istanbul Aydin University, VM Medical Park Florya Hospital, Istanbul, Türkiye; ^2^ Department of Radiology, Kocaeli City Hospital, Kocaeli, Türkiye

**Keywords:** artificial intelligence, patient education, varicocele embolization, interventional radiology, empathy, academic accuracy, large language models

## Abstract

**Background:**

Large language models (LLMs) appear to be capable of performing a variety of tasks, including answering questions, but there are few studies evaluating them in direct comparison with clinicians. This study aims to compare the performance of artificial intelligence (AI) models and clinical specialists in informing patients about varicocele embolization. Additionally, we aim to establish an evidence base for future hybrid informational systems that integrate both AI and clinical expertise.

**Methods:**

In this prospective, double-blind, randomized controlled trial, 25 frequently asked questions about varicocele embolization (collected via Google Search trends, patient forums, and clinical experience) were answered by three AI models (ChatGPT-4o, Gemini Pro, and Microsoft Copilot) and one interventional radiologist. Responses were randomized and evaluated by two independent interventional radiologists using a valid 5-point Likert scale for academic accuracy and empathy.

**Results:**

Gemini achieved the highest mean scores for both academic accuracy (4.09 ± 0.50, 95% CI: 3.95–4.23) and higher expert-rated scores for empathetic communication (3.54 ± 0.59, 95% CI: 3.38–3.70), followed by Copilot (academic: 4.07 ± 0.46, 95% CI: 3.94–4.20; empathy: 3.48 ± 0.53, 95% CI: 3.33–3.63), ChatGPT (academic: 3.83 ± 0.58, 95% CI: 3.67–3.99; empathy: 2.92 ± 0.78, 95% CI: 2.70–3.14), and the comparator physician (academic: 3.75 ± 0.41, 95% CI: 3.64–3.86; empathy: 3.12 ± 0.82, 95% CI: 2.89–3.35). ANOVA revealed statistically significant differences across groups for both academic accuracy (F = 6.181, *p* < 0.001, *η*^2^ = 0.086) and empathy (F = 9.106, *p* < 0.001, *η*^2^ = 0.122). Effect sizes were medium for academic accuracy and large for empathy.

**Conclusions:**

AI models, particularly Gemini, received higher ratings from expert evaluators compared to the comparator physician in patient education regarding varicocele embolization, excelling in both academic accuracy and empathetic communication style. These preliminary findings suggest that AI models hold significant potential to complement patient education systems in interventional radiology practice and provide compelling evidence for the development of hybrid patient education models.

## Introduction

The rapid advancement of artificial intelligence (AI) technologies has initiated significant evolution in medical practice, particularly in patient education and consultation processes (1). Large language models (LLMs) such as ChatGPT, Gemini, and Copilot, demonstrate remarkable performance in generating detailed and coherent responses to medical inquiries (2, 3). These advancements significantly enhance physician–patient communication, enabling more informative and effective interactions (4). Through the contributions of LLMs, healthcare professionals can improve the clarity of shared information, thereby allowing patients to make more informed decisions about their health and participate more actively in diagnostic and therapeutic processes (5). However, their performance in terms of medical accuracy, reliability, and empathetic communication—especially within the context of specialized interventional procedures—remains insufficiently explored (3).

Varicocele embolization has emerged as a minimally invasive approach for the treatment of varicocele, one of the leading causes of male infertility (6). Compared to surgical approaches, this treatment provides lower morbidity, shorter recovery times, and higher technical success rates (7). However, public awareness and the accessibility of the procedure remain limited (8). Patients frequently present with numerous questions regarding the treatment; therefore, accurate and comprehensible patient education is essential to enhance patient satisfaction, ensure treatment adherence, and improve clinical outcomes (9).

Traditional patient examination and information delivery methods face considerable limitations due to physicians' heavy workload, limited time, and inconsistencies in information transfer (10). AI-assisted patient education systems may provide innovative solutions to these challenges by offering continuous accessibility, up-to-date and standardized information, multilingual support, and consistent responses regardless of physician fatigue or availability (11).

Patient education processes consist of several critical components. Among these, academic accuracy and empathy stand out as the most important. Academic accuracy refers to the provision of information that is current, scientifically validated, and aligned with clinical guidelines (12). Empathy, in contrast, involves addressing patients' emotional needs, offering reassurance, and fostering supportive communication (13). These two elements are crucial for patient satisfaction, treatment adherence, and favorable clinical outcomes, forming the foundation of modern patient-centered care (14).

This study aims to objectively compare the AI models and the physicians in varicocele embolization patient education with respect to academic accuracy and empathy, thereby systematically evaluating the potential advantages and limitations of AI-assisted patient education.

## Materials and methods

### Study design and ethics

This prospective, double-blind, randomized controlled analysis was designed to evaluate responses to patient questions with respect to varicocele embolization education provided from different sources. This study was conducted in accordance with the principles of the Declaration of Helsinki. Comprehensive randomization and blinding protocols were implemented to minimize pattern bias and assessor-related bias.

### Question selection and categorization

Twenty-five frequently asked questions about varicocele embolization were identified through the following: a systematic literature review, patient forum analyses, the most common queries from Google searches, and a decade of clinical experience. The complete list of 25 questions, the exact prompts used for the AI models, and the full verbatim responses from all four sources are available in both Turkish and English in the Supplementary Material. The questions were categorized into five evidence-based domains:

• **General Information and Treatment Options (Q1–Q5):** Efficacy, advantages/disadvantages and comparisons with alternative treatments
• **Procedural Details (Q6–Q10):** Workflow, anesthesia, pain management, and preparation protocols
• **Efficacy and Outcomes (Q11–Q15):** Success rates, sperm quality, testosterone levels, and fertility outcomes
• **Risks and Complications (Q16–Q20):** Potential risks, adverse effects, safety profile, and contraindications
• **Recovery Process (Q21–Q25):** Post-procedure care, follow-up protocols, and lifestyle recommendations

### Response sources and standardization

Responses were collected from four distinct sources using standardized protocols. ChatGPT-4o (OpenAI), Gemini Pro (Google), and Microsoft Copilot generated the answers, while the comparator physician is a board-certified interventional radiologist with five years of experience, performing more than 40 varicocele embolizations annually. All AI models were queried using identical prompt formats, and responses were transcribed verbatim without modifications.

The comparator physician, a board-certified interventional radiologist with five years of experience, was instructed to answer the 25 questions based on their clinical expertise and general knowledge, without consulting external resources or guidelines. This was designed to simulate a typical, real-time patient consultation scenario. No time constraints were imposed on the physician for generating the responses.

### Randomization and blinding protocol

A multilayered randomization protocol was implemented to minimize bias. For each question, four answers were randomized as options A, B, C, and D, with evaluators blinded to their sources. Each source appeared approximately equally across all positions (A–D), and unique randomization was applied to each question. Both evaluators and the statistician were blinded to response sources. The randomization table was stored separately and kept concealed until the analysis phase.

### Evaluation criteria and validation

Each response was independently evaluated across two domains—academic accuracy and empathy—using a 5-point Likert scale (15). For academic accuracy, a score of 5 indicated completely accurate, guideline-consistent, and comprehensive responses; 4 denoted mostly accurate responses with minor omissions; 3 reflected partially accurate responses with some errors; 2 indicated largely inaccurate responses with significant omissions; 1 represented completely inaccurate responses containing potentially harmful information. For empathy, a score of 5 indicated highly empathetic, reassuring, and supportive communication; 4 represented empathetic and understanding interaction; 3 denoted a neutral approach with moderate empathy; 2 indicated limited empathy and cold communication; 1 reflected robotic, unsympathetic, and impersonal responses.

### Evaluators and reliability

**Figure 1:**
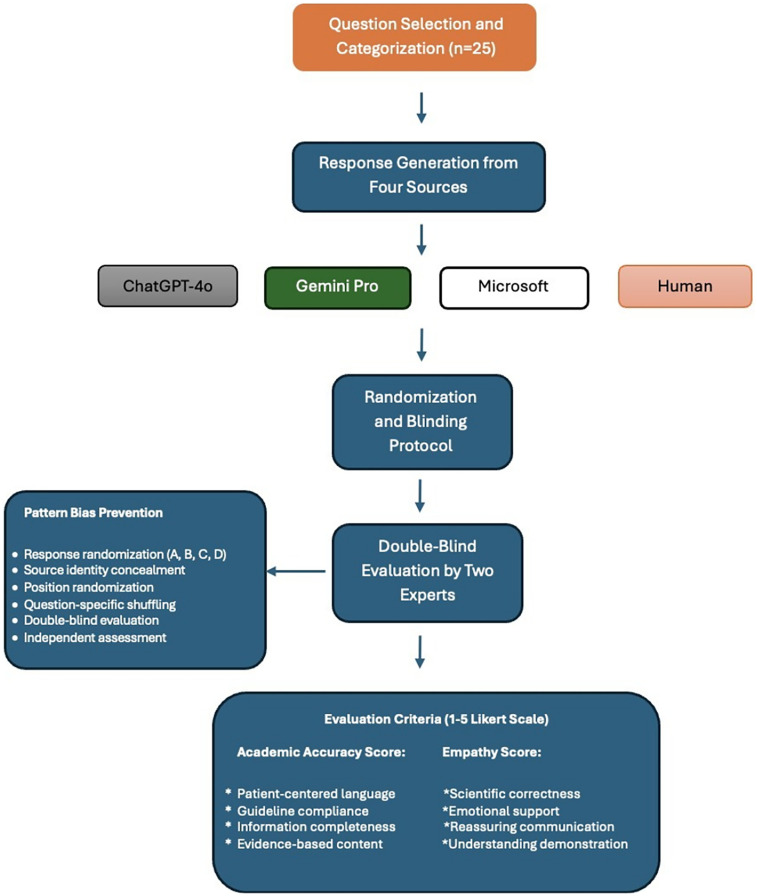
Flowchart of study methodology.

Evaluations were conducted by two independent interventional radiologists, each with over 5 years of experience and having performed more than 30 varicocele embolizations annually. Both evaluators received prior orientation with detailed definitions and examples of the Likert scale categories to ensure consistency in scoring. The study methodology is summarized in Figure 1.

### Statistical analysis

Data were analyzed using SPSS 28.0. The sample size was determined via power analysis assuming α = 0.05, β = 0.20, and a medium effect size (f = 0.25). Statistical methods included descriptive statistics (mean, standard deviation, median, interquartile range, and 95% confidence intervals), normality testing (Shapiro–Wilk and Kolmogorov–Smirnov); homogeneity of variances (Levene's and Brown-Forsythe tests), group comparisons (one-way ANOVA and Welch's ANOVA), *post hoc* analyses (Tukey's HSD and Games Howell); pairwise comparisons (independent-samples *t*-tests and Mann–Whitney *U*-tests); effect size calculations (eta-squared [*η*^2^] and Cohen's d); and reliability analyses (Pearson correlation coefficient and intraclass correlation coefficients [ICC]). A *p*-value <0.05 was considered statistically significant, with Bonferroni corrections applied for multiple comparisons.

## Results

### Participant characteristics and descriptive statistics

A total of 100 responses (25 questions × 4 sources) were evaluated. Each source provided 25 responses, and a comprehensive statistical analysis was performed. Detailed descriptive statistics are presented in Table 1.

**Table 1:** Source-based descriptive statistics.

Source	Academic accuracy					Empathy				
	Mean ± SD	Min–Max	Med	IQR	95% CI	Mean ± SD	Min–Max	Med	IQR	95% CI
Gemini	4.09 ± 0.50	3.0–5.0	4.0	3.5–4.5	3.95–4.23	3.54 ± 0.59	2.0–4.5	3.5	3.0–4.0	3.38–3.70
Copilot	4.07 ± 0.46	3.0–5.0	4.0	3.5–4.5	3.94–4.20	3.48 ± 0.53	2.5–4.5	3.5	3.0–4.0	3.33–3.63
ChatGPT	3.83 ± 0.58	2.5–5.0	4.0	3.5–4.5	3.67–3.99	2.92 ± 0.78	1.5–4.0	3.0	2.25–3.75	2.70–3.14
Comparator Physician	3.75 ± 0.41	2.5–4.5	3.5	3.0–4.0	3.64–3.86	3.12 ± 0.82	1.0–4.5	3.0	2.5–3.5	2.89–3.35

SD, standard deviation; Med, median; IQR, interquartile range; CI, confidence interval.

### Statistical comparisons and hypothesis testing

#### Normality and homogeneity of variances

The Shapiro–Wilk tests indicated deviations from normality in some groups (*p* < 0.05). Levene's test confirmed the homogeneity of variances (academic accuracy: *p* = 0.795; empathy: *p* = 0.948). Considering the robustness of ANOVA and the central limit theorem, parametric analyses were conducted.

ANOVA Results: One-way ANOVA revealed statistically significant differences among the groups with respect to both academic accuracy (F(3, 196) = 6.181, *p* < 0.001, *η*^2^ = 0.086) and empathy scores (F(3, 196) = 9.106, *p* < 0.001, *η*^2^ = 0.122). Full statistical comparisons are presented in Table 2.

**Table 2:** Results of statistical comparisons.

Analysis	Academic accuracy	Empathy
ANOVA		
F value	6.181	9.106
Degrees of freedom	3, 196	3, 196
*p* value	<0.001*	<0.001*
95% CI for F	2.65–9.71	4.12–14.10
Effect size		
*η*^2^ (Eta-squared)	0.086	0.122
95% CI for *η*^2^	0.02–0.17	0.04–0.21
Effect level	Medium	Large
Power analysis		
Observed power	0.95	0.98
Levene's test		
W value	1.245	2.156
*p* value	0.295	0.095

* *p* < 0.05 considered statistically significant.

**Figure 2:**
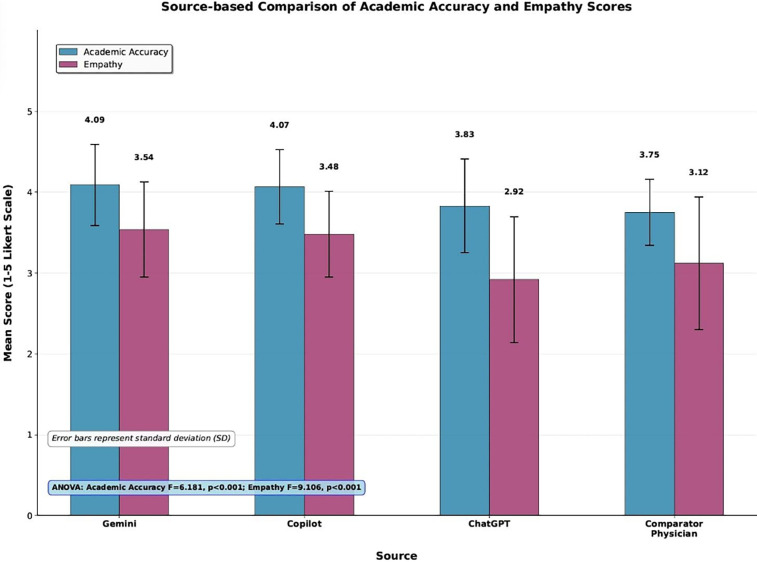
Source-based comparison of academic accuracy and empathy scores. Bar chart showing mean scores with standard deviation error bars for AI models (Gemini, Copilot, ChatGPT) and the comparator physician across academic accuracy and empathy metrics.

A detailed comparison of academic accuracy and empathy scores across sources is illustrated in Figure 2.

### Post hoc analyses and pairwise comparisons

Following significant ANOVA results, Tukey HSD *post hoc* tests were conducted, and after Bonferroni correction, several pairwise comparisons reached statistical significance. In terms of academic accuracy, *post-hoc* tests revealed that Gemini (*p* < 0.001) and Copilot (*p* = 0.002) scored significantly higher than the comparator physician. Gemini also scored significantly higher than ChatGPT (*p* = 0.021). A similar trend was observed for empathy, where Gemini (*p* < 0.001) and Copilot (*p* = 0.001) significantly outperformed the comparator physician. Gemini also scored significantly higher than ChatGPT (*p* < 0.001) and the comparator physician (*p* < 0.001). Copilot also scored significantly higher than ChatGPT (*p* < 0.001) and the comparator physician (*p* = 0.001). Overall, these findings demonstrate Gemini's superior performance as rated by expert evaluators in both academic accuracy and empathy.

### Inter-rater reliability and consistency

Analyses of inter-rater agreement revealed the Pearson correlation coefficient was not significant for academic accuracy (*r* = 0.038, *p* = 0.773, 95% CI: −0.235 to 0.307), and showed borderline significance was observed for empathy (*r* = 0.249, *p* = 0.056, 95% CI: −0.007 to 0.477). The intraclass correlation coefficient (ICC) values indicated poor agreement for academic accuracy (ICC = 0.032, 95% CI: −0.156 to 0.218) and modest agreement for empathy (ICC = 0.256, 95% CI: 0.021 to 0.467). These results suggest notable differences in evaluators' perspectives.

### Category-based performance analysis

In the category-based analysis, Gemini ranked first in general information, efficacy and outcomes, and recovery process, second in procedural details, and second to the comparator physician in the risks category. Overall, Gemini led in four of the five categories, confirming its overall superior performance as rated by expert evaluators.

## Discussion

A significant finding of this study is that AI models, particularly Gemini, received higher ratings from expert evaluators compared to the comparator physician in both academic accuracy and empathetic communication style regarding varicocele embolization patient education. Supported by large effect sizes (Cohen's d > 0.8), these results are consistent with an emerging body of evidence (16, 17) comparing AI and clinical specialist performance in medical communication. They suggest that AI-assisted systems may herald a significant evolution in patient education, with the potential to transform traditional physician–patient communication.

Gemini's superior performance may be attributed to several factors. Google's access to vast and diverse datasets, including a significant corpus of medical literature, likely provides a robust knowledge base. Furthermore, its underlying architecture, such as the Pathways Language Model 2 (PaLM 2), is designed for advanced reasoning and nuanced language understanding, which may contribute to its higher scores in both academic accuracy and generating responses perceived as empathetic by experts (18).

This finding, while notable, is consistent with an emerging body of evidence suggesting that AI can generate responses that are rated as highly empathetic by human evaluators (11, 17). This study adds to the findings of recent research (17), which also reported high ratings for AI in empathetic communication, suggesting that our results confirm a growing trend in the literature. This suggests that the concept of empathy in patient education may require redefinition. Several theoretical explanations can be proposed: AI models can consistently provide empathetic responses that are unaffected by fatigue or stress. They employ communication strategies optimized for patient-centered care, including empathetic expressions. Moreover, unlike humans, they are free from unconscious biases that may reduce empathetic engagement with certain patient groups (19–21).

A notable finding is the low inter-rater reliability for both academic accuracy and empathy. This discrepancy may stem from the inherent subjectivity in evaluating communication, particularly empathy. Despite the orientation session, individual evaluators may have different internal standards and interpretations. For instance, one evaluator might prioritize a reassuring tone, while another might focus on the technical completeness of the answer. This highlights the challenge of standardizing the assessment of qualitative metrics in medical communication and suggests that future studies could benefit from more rigorous calibration methods or the inclusion of a third evaluator to adjudicate disagreements.

The superior academic accuracy of AI models holds substantial implications for patient counseling. This advantage may stem from their access to the comprehensive medical literature, their ability to rapidly synthesize up-to-date knowledge, and their immunity to human error (22). Gemini's performance, in particular, reflects Google's strategic investments in healthcare-focused AI, including the development of specialized medical models such as Med-PaLM (23).

Previous comparative studies have demonstrated substantial variability in the performance of large language models depending on the medical domain. Consistent with the findings of Demir et al. in their keratoconus study (24), our results confirm that performance differences exist among models and are influenced by domain specificity.

Although interventional radiology often achieves outcomes comparable to or superior to surgical procedures, it remains relatively underrecognized among patients (25). Through minimally invasive techniques, interventional radiology offers shorter recovery times, lower complication rates, and higher patient satisfaction (26). In this context, AI-assisted patient education systems may play a critical role by offering evidence-based and unbiased information, preventing referrals exclusively to surgical specialties, supporting informed decision-making, and enhancing public awareness of interventional radiology.

The integration of artificial intelligence (AI) into patient education raises a set of ethical and practical issues, including the preservation of the human element within the physician–patient relationship; the limitations of current technologies that cannot replace uniquely human capacities such as empathy and the interpretation of nonverbal cues; and the question of accountability when erroneous or incomplete information is produced, which underscores the need for clear legal and regulatory frameworks. In addition, the privacy, security, ownership, and consent surrounding the sensitive health data generated by AI interactions; inequities arising from differences in digital literacy, language, and access to technology; biases inherent in models trained on specific populations; and the expansion of informed consent to explicitly cover the role, limitations, and error potential of AI are priority domains that demand transparent communication.

From the perspective of professional autonomy and competence, excessive reliance on AI may increase the risk of deskilling, whereas appropriately designed systems can reduce routine workload and free time for clinical reasoning and patient interaction; striking the balance is essential. Patients accustomed to receiving “instant and comprehensive” responses from AI may also develop unrealistic expectations, potentially straining the physician–patient relationship. Accordingly, hybrid models of care—anchored in human oversight, transparency, continuous quality and safety monitoring, bias mitigation, and clearly delineated lines of responsibility—should be pursued. Robust ethical guidelines and professional standards, reinforced by ongoing dialogue among clinicians, ethicists, policymakers, and patients, will ensure that AI augments rather than replaces compassionate, person-centered, and individualized care.

AI-driven pre-consultation systems carry transformative potential for the future of healthcare. By generating personalized pre-visit reports that include patient history, concerns, and complex unresolved questions, these systems can reduce the time physicians spend on routine questioning, thereby allowing them to focus more on empathetic communication and patient-centered evaluations.

Based on our findings, we propose a hybrid three-phase hybrid model for patient education. In the first phase, patients use AI systems to address basic pre-procedure questions, with comprehension and satisfaction measured, while complex or atypical concerns are escalated to physicians. In the second phase, the physician addresses unresolved questions, evaluates the patient's individual context, and finalizes clinical decision-making. In the third phase, post-procedure queries are managed by AI, with escalation protocols in place for emergencies.

The proposed approach offers several advantages, including 24/7 accessibility, consistent quality of information and empathy, improved time efficiency, and cost-effectiveness. However, risks such as the potential dissemination of incorrect information, diminished physician–patient rapport, and possible oversight of complex cases must be considered. To mitigate these risks, continuous content updates, hybrid implementation strategies, and structured escalation protocols are recommended.

While this study provides valuable insights, its findings are primarily situated within the context of interventional radiology and varicocele embolization. The principles of AI-assisted patient education may be applicable to other procedural specialties such as cardiology, oncology, or general surgery. However, the specific content and communication style would need to be adapted and validated for each clinical domain. Therefore, the direct generalizability of these findings to other areas of medicine requires further investigation.

Future projections and the strategic importance of interventional radiology may be an important points to acknowledge. Given the challenges of limited patient awareness and accessibility, AI-assisted patient education systems may be strategically vital for interventional radiology. They have the potential to enhance specialty visibility, facilitate equitable access to treatment options, and provide unbiased, evidence-based education.

## Study limitations and future directions

This study has several important limitations. First and foremost, this study did not involve real patients. The evaluation of academic accuracy and empathy was conducted by expert radiologists, and their ratings may not reflect the actual patient experience, satisfaction, or comprehension. This represents a significant limitation. Second, our comparison involved a single comparator physician. This limits the generalizability of our findings to the broader clinician population, as communication style and knowledge can vary significantly among individuals. Third, the study was conducted using Turkish-language responses, which may limit the generalizability of the findings. The performance of LLMs and the perception of empathy can vary significantly across different languages and cultural contexts. The scope of the 25 selected questions may not capture the full range of patient concerns regarding varicocele embolization. The focus on a single specialty, reliance on only two evaluators, and potential influence of cultural factors due to the use of Turkish-language assessments may limit generalizability. Furthermore, the absence of real patient interaction means that the laboratory setting may not fully reflect clinical practice.

Future studies should prioritize prospective, multicenter designs involving actual patients to directly measure patient satisfaction, understanding, and clinical outcomes. To enhance reliability, a larger and more diverse panel of pre-calibrated evaluators should be used; when necessary, scoring discrepancies should be resolved by a third evaluator. To test the generalizability of findings, similar protocols should be applied in different languages and healthcare systems, as well as in other specialties such as cardiology, oncology, and neurology. Long-term follow-up and cost-effectiveness analyses should be added for a more robust measurement of clinical impact. In the current study, responses were not evaluated by actual patients in terms of empathy and comprehensibility; we plan to address this gap with patient-based evaluations in our future studies.

## Conclusion

This study demonstrated that AI models, particularly Gemini, received higher expert ratings than the comparator physician in patient education for varicocele embolization in terms of both academic accuracy and empathetic communication style. These preliminary findings indicate that we are witnessing a significant evolution in patient education, with AI-assisted systems poised to play a transformative role in medical practice.

AI-based pre-consultation systems should be regarded as an innovative, evidence-based advancement in both patient education and clinical decision support, particularly in specialties such as interventional radiology, where patient awareness remains limited despite high clinical efficacy.

Hybrid models appear to be the most suitable approach, combining the proven advantages of AI with the indispensable human touch, thereby ensuring both efficiency and compassionate care. The widespread adoption of AI in patient education is expected in the near future. Throughout this process, patient safety, ethical principles, and clinical effectiveness must remain top priorities. With ongoing research and development, the full potential of these technologies can be realized, ultimately resulting in systematic improvements in with respect to the quality of patient care.

## Data Availability

The raw data supporting the conclusions of this article will be made available by the authors, without undue reservation.
