# Supplementary material for: Comparison of artificial intelligence models and physicians in patient education for varicocele embolization: a double-blind randomized controlled trial
Source: Front Radiol. 2025 Oct 14;5:1682725. doi: 10.3389/fradi.2025.1682725 (PMC12558931; doi:10.3389/fradi.2025.1682725)
Supplement: Supplementary file 1 [file Datasheet1.pdf]

QUESTIONS AND ANSWERS: Answers to the 25 most frequently asked questions about varicocele embolization by Artificial Intelligence and Human (Interventional Radiologist).

CHAT GPT

SECTION 1: GENERAL INFORMATION AND TREATMENT OPTIONS

1. **Is embolization a good treatment for varicocele?**
   Yes, embolization is a minimally invasive method and an effective treatment option for most patients.
2. **What are the advantages and disadvantages of varicocele embolization?**
   Advantages: Non-surgical, fast recovery, local anesthesia. Disadvantages: Need for radiologic equipment, risk of recurrence, not suitable for every patient.
3. **Which varicocele treatment is best: microsurgery or embolization?**
   Microsurgery has lower recurrence and higher success rates. However, embolization is less invasive. It varies according to patient selection.
4. **What happens if varicocele is not treated?**
   It can lead to problems such as infertility, shrinkage in testicular volume, and pain.
5. **What are the ways to treat varicocele without surgery?**
   Embolization, lifestyle changes, using supportive underwear, and cold applications.

SECTION 2: PROCEDURE DETAILS

6. **Are you awake during the embolization procedure?**
   Yes, during the procedure the patient is usually under local anesthesia and is awake.
7. **Is embolization a painful procedure?**
   Usually a mild discomfort is felt during the procedure; severe pain is rare.
8. **How long does the embolization procedure take?**
   It usually lasts between 30 and 60 minutes.
9. **How should one prepare for embolization?**
   It is necessary to come fasting, have blood tests performed, and report allergy history.

10. **Is embolization considered a major operation?**
    No, embolization is a minimally invasive intervention; it does not fall into the major surgery class.

SECTION 3: EFFECTIVENESS AND OUTCOMES

11. **What is the success rate of varicocele embolization?**
    It has a technical success rate of around 85–95%.
12. **Does sperm quality increase after varicocele embolization?**
    Yes, in most men an increase in sperm count and motility is observed.
13. **Does testosterone level increase after embolization?**
    A slight increase may occur in some patients, but it is not always evident.
14. **How long is varicocele embolization effective?**
    It can be effective for years, but the probability of recurrence is around 10–20%.
15. **Does embolization increase fertility?**
    Yes, especially in men with impaired sperm parameters, fertility rates may increase.

SECTION 4: RISKS AND COMPLICATIONS

16. **Can complications occur during varicocele embolization??**
    Although rare, there is a risk of vessel injury, infection, or failure.
17. **Can varicocele embolization fail?**
    Yes, in 5–10% the vein may not be completely occluded or may reopen.
18. **What are the long-term side effects of varicocele embolization?**
    Serious long-term side effects are rare; some patients may have mild chronic pain.
19. **Can varicocele return after embolization?**
    Yes, it may recur at a rate of 10–20%.
20. **Can embolization cause infertility?**
    No, when done correctly it does not create a risk of infertility; it usually increases fertility.

SECTION 5: RECOVERY PROCESS

21. **How long does recovery take after varicocele embolization?**
    Most patients return to normal life within 1–2 days.

22. **When can I walk after embolization?**
    You can walk a few hours after the procedure.
23. **How should one sleep after embolization?**
    It is recommended to sleep on the back or in a slightly elevated position for the first few days.
24. **When can I go to the gym after embolization?**
    Usually, light exercises can be started after 1 week; for heavy sports, 2–3 weeks are waited.
25. **What happens to the veins after embolization?**
    The treated veins close and, over time, are absorbed by the body or shrink.

GEMINI

SECTION 1: GENERAL INFORMATION AND TREATMENT OPTIONS

1. **Is embolization a good treatment for varicocele?**
   Yes, embolization is a good and minimally invasive treatment option for varicocele. It is regarded as a good alternative to surgery and usually offers a faster recovery process.
2. **What are the advantages and disadvantages of varicocele embolization?**
   Advantages:
   • Minimally Invasive: No large incision is made; only a small needle hole is sufficient.
   • Faster Recovery: Compared with surgery, the recovery period is usually shorter.
   • Outpatient Treatment: Most of the time, hospitalization is not required; you can return home the same day.
   • Less Pain: Post-procedure pain is generally milder than surgery.
   • Local Anesthesia: It is usually performed under local anesthesia; there are no general-anesthesia risks.
   • Applicability to Both Sides: It can be used in both unilateral and bilateral varicoceles.

Disadvantages:
• Risk of Recurrence: Even if rarely, varicocele may recur.
• Radiation Exposure: A certain amount of radiation is used during the procedure.
• Contrast Agent Reaction: Allergic reactions may rarely be seen.

• Limited Experience: A specialist with sufficient experience may not be available in every center.

3. **Which varicocele treatment is best: microsurgery or embolization?**

The best treatment depends on the individual situation. Both microsurgery and embolization are effective treatment methods. While microsurgery may have a slightly lower recurrence rate, embolization stands out by being less invasive and offering faster recovery. You should decide which method is more suitable for you by talking with your doctor.

4**. What happens if varicocele is not treated?**

• Untreated varicocele may progress and lead to the following problems:
• Infertility: It can negatively affect sperm quality and count.
• Testicular Atrophy (Shrinkage): It may cause a reduction in testicular size.
• Pain: Chronic or recurrent testicular pain may be seen.
• Low Testosterone: It may lead to a decrease in hormone levels.

5. What are the ways to treat varicocele without surgery?

Embolization is the main way to treat varicocele without surgery. Apart from this, there is no other non-surgical method that directly eliminates varicocele. In some cases, symptomatic treatments or lifestyle changes may be recommended for pain management, but these do not treat varicocele.

SECTION 2: PROCEDURE DETAILS

6. **Are you awake during the embolization procedure?**
   Yes, you are usually awake during embolization. The procedure is performed under local anesthesia, so the area where the procedure is performed becomes numb. If needed, mild sedation (a tranquilizer) may also be given.
7. **Is embolization a painful procedure?**
   No, embolization is generally not a painful procedure. Since local anesthesia is applied, apart from the first moment when the needle is inserted, severe pain is not felt. A mild pressure or discomfort may be felt during the procedure.
8. **How long does the embolization procedure take?**
   Varicocele embolization generally takes between 30 minutes and 1 hour. However, this period may vary depending on the complexity of the varicocele and the patient's condition.

9. **How should one prepare for embolization?**
   • Preparation for embolization generally includes the following:
   • Fasting: You are asked to fast for a certain period (usually 6–8 hours) before the procedure.
   • Medications: You must inform your doctor of all medications you use. Blood-thinning medications may need to be stopped before the procedure.
   • Fluid Intake: Adequate fluid intake is important before the procedure.
   • Clothing: It is recommended to wear comfortable clothing.
   • Tests: Blood tests and other imaging tests may be requested before the procedure.
10. **Is embolization considered a major operation?**
    No, embolization is not considered a major operation. It is a minimally invasive intervention and, compared to traditional open surgery, it is a method that is much less risky and has a faster recovery process. It is generally referred to as an "interventional radiological procedure" rather than "surgery."

SECTION 3: EFFECTIVENESS AND OUTCOMES

11. **What is the success rate of varicocele embolization?**
    The success rate of varicocele embolization is quite high, generally around 90–95%. This rate is evaluated in terms of the varicocele being completely closed and the symptoms improving.
12. **Does sperm quality increase after varicocele embolization?**
    Yes, an increase in sperm quality may be seen after varicocele embolization. Its positive effects on sperm count, motility, and morphology have been shown by scientific studies. However, the same degree of improvement may not be seen in every case.
13. **Does testosterone level increase after embolization?**
    Yes, some studies have observed an increase in testosterone levels after embolization. With the elimination of the negative effect of varicocele on testosterone production, hormone levels may return to normal or improve.
14. **How long is varicocele embolization effective?**
    Varicocele embolization aims to provide a permanent effect. The varicose veins that are successfully closed generally remain closed. However, even if rarely, the

formation of a new varicocele through different veins or the recurrence of the existing varicocele is possible.

15. **Does embolization increase fertility?**
    Yes, embolization can help increase fertility in men who suffer from infertility. Varicocele can lead to infertility by disrupting sperm production due to heat increase and blood pooling. Embolization eliminates these adverse effects and can increase the chance of pregnancy either naturally or with assisted reproductive techniques.

SECTION 4: RISKS AND COMPLICATIONS

16. **Can complications occur during varicocele embolization??**
    As with any medical procedure, complications may rarely occur in embolization, but these are generally mild. “Going wrong” refers to small complications rather than a major problem. These include bruising at the injection site, mild pain, or allergic reactions. Serious complications are very rare.
17. **Can varicocele embolization fail?**
    Yes, although rarely, varicocele embolization may fail. Failure may present as the veins not being completely occluded or the varicocele recurring shortly after. In this case, another treatment method or repeat embolization may be considered.
18. **What are the long-term side effects of varicocele embolization?**
    Known serious long-term side effects of varicocele embolization are very rare. The most common long-term concern is the possibility of recurrence of the varicocele. Other potential but rare effects include chronic pain or testicular injury, but these are exceptional.
19. **Can varicocele return after embolization?**
    Yes, although rarely, varicocele may return (recur) after embolization. This usually occurs because of veins that were not completely occluded during embolization or the dilation of new veins over time. The recurrence rate may be slightly higher than with microsurgery, but it is still low.
20. **Can embolization cause infertility?**
    No, varicocele embolization does not cause infertility. On the contrary, it is a method that aims to treat infertility caused by varicocele and to increase fertility.

SECTION 5: RECOVERY PROCESS

21. **How long does recovery take after varicocele embolization?**
    Recovery after varicocele embolization is usually very fast. Most patients can return to normal activities a few hours after the procedure. Full recovery and relief are completed within a few days. It is recommended to stay away from heavy physical activities for a few days.
22. **When can I walk after embolization?**
    You can usually walk immediately after embolization. After a short rest period (about 1–2 hours) following the procedure, there is no harm in getting up and walking.
23. **How should one sleep after embolization?**
    There is no requirement for a special sleep position after embolization. You can sleep in the position in which you feel most comfortable. Since there may be mild tenderness at the procedure site, it is preferable to lie in a way that does not put pressure on this area.
24. **When can I go to the gym after embolization?**
    The return time to the gym after embolization is usually a few days. While light exercises can be started 2–3 days later, it is recommended to wait about 1 week for heavy lifting or intense sports activities. Your doctor will give you personalized recommendations.
25. **What happens to the veins after embolization?**
    After embolization, the varicose veins that cause varicocele are permanently closed and shrink by contracting over time. These veins no longer carry blood, and blood flow continues through healthy veins. The body absorbs these veins over time.

COPILOT

SECTION 1: GENERAL INFORMATION AND TREATMENT OPTIONS

1. **Is embolization a good treatment for varicocele?**
   Yes, embolization is an effective and safe non-surgical option in the treatment of varicocele. It is especially suitable for those seeking an alternative to surgery. The success rate is around 85–90%, and in most cases it reduces symptoms and improves sperm quality.
2. **What are the advantages and disadvantages of varicocele embolization?**
   Advantages:
   • It is non-surgical: Access is only through a vein; there is no incision.

• It is performed with local anesthesia; you are usually awake.

• Fast recovery: You can return to normal life within 1–2 days.

• Lower risk of complications: Surgical risks such as infection and testicular injury are low.

• If there is bilateral varicocele, both can be treated in a single session.

Disadvantages:

• It is not performed in every center; an interventional radiology specialist is required.

• A recurrence rate of 10–15% may be seen.

• Embolization material may rarely migrate.

• Temporary pain or discomfort may occur in some people.

3. **Which varicocele treatment is best: microsurgery or embolization?**

   Both methods are effective. Comparison:

**Feature**	**Microsurgery**	**Embolization**
Anesthesia	General/spinal	Local
Recovery time	2–3 weeks	1–2 days
Incision	Present	None
Recurrence rate	Around 10%	Around 10–15%
Bilateral treatment	Two separate incisions	Can be done in one session

Conclusion: Embolization is more comfortable; microsurgery may offer a lower recurrence rate in some cases.

4. **What happens if varicocele is not treated?**

Untreated varicocele:

• May impair sperm production

• May lead to testicular shrinkage (atrophy)

• May cause problems such as low testosterone, decreased sexual desire, and erectile dysfunction

• The risk of infertility increases

5. **What are the ways to treat varicocele without surgery?**

• Embolization: The most effective non-surgical method.

• Lifestyle changes: Exercise, avoiding tight clothing, staying away from hot showers.

• Antioxidant supplements: Vitamin C, zinc, coenzyme Q10.

• Herbal supports: Although scientific evidence is limited, some may support circulation.

SECTION 2: PROCEDURE DETAILS

6. **Are you awake during the embolization procedure?**
   Yes, local anesthesia and mild sedation are usually applied. You are awake, but thanks to relaxing medications you do not feel pain during the procedure.
7. **Is embolization a painful procedure?**
   No, it is not painful. Only a mild pressure or warmth may be felt at the entry site. Afterwards, mild groin pain for a few days may occur but is usually controlled with simple painkillers.
8. **How long does the embolization procedure take?**
   The procedure time is usually between 30 and 60 minutes. You can be discharged the same day.
9. **How should one prepare for embolization?**
   • If you take blood-thinning medications, you should consult your doctor.
   • Fasting for 4–6 hours before the procedure may be necessary.
   • Comfortable clothes should be worn.
   • You should have someone with you to take you home.
10. **Is embolization considered a major operation?**
    No, it is a minimally invasive procedure. There is no incision, and hospitalization is not required. It is usually done as a day case.

SECTION 3: EFFECTIVENESS AND OUTCOMES

11. **What is the success rate of varicocele embolization?**
    The success rate is around 85–90%. It provides a marked improvement in sperm parameters and pain.
12. **Does sperm quality increase after varicocele embolization?**
    • Sperm count, motility, and morphology may improve.
13. **Does testosterone level increase after embolization?**
    • A mild increase in testosterone level may be seen.
    • The effect usually appears within 3 months.

14. **How long is varicocele embolization effective?**

    The effect of a successful embolization is permanent. However, in some cases there may be recurrence and re-treatment may be required.

15. **Does embolization increase fertility?**

    Yes, especially in men with impaired sperm parameters, an increase in fertility may be observed. In some couples, pregnancy rates rise.

SECTION 4: RISKS AND COMPLICATIONS

16. **Can complications occur during varicocele embolization??**

    Although rare:

    • The embolization material may go to the wrong place

    • Infection, allergy, or bleeding may occur

    • The procedure may fail

17. **Can varicocele embolization fail?**

    Yes, a recurrence rate of 10–15% may be experienced.

18. **What are the long-term side effects of varicocele embolization?**

    • Serious long-term side effects are rare.

    • Some people may have mild testicular pain or venous sclerosis.

19. **Can varicocele return after embolization?**

    Yes, it may reoccur in some cases.

20. **Can embolization cause infertility?**

    Generally no. The goal is to increase fertility; the risk of infertility is quite low.

SECTION 5: RECOVERY PROCESS

21. **How long does recovery take after varicocele embolization?**

    You can return to normal life within 1–2 days.

22. **When can I walk after embolization?**

    You can usually walk on the same day.

23. **How should one sleep after embolization?**

    It is recommended to sleep on your back and in a comfortable position.

24. **When can I go to the gym after embolization?**

    Usually you can exercise after 1 week.

25. **What happens to the veins after embolization?**
    The embolized veins close, and blood flow is directed to healthy veins. This reduces the pressure in the testis and corrects symptoms.

HUMAN

SECTION 1: GENERAL INFORMATION AND TREATMENT OPTIONS

1. **Is embolization a good treatment for varicocele?**
   Yes, because in this disease the diseased veins there need to be closed.
2. **What are the advantages and disadvantages of varicocele embolization?**
   Advantages include that it can be done without hospitalization, it is approximately a half-hour to 45-minute procedure, and the risk of complications is very low; although there are not really many disadvantages, radiation and contrast-agent use can be counted.
3. **Which is better, microsurgery or embolization?**
   Although studies do not show a superiority of one procedure over the other, as an interventional radiologist, if I had a varicocele I would prefer embolization treatment, because I would prefer treatment performed through a small needle hole without surgery.
4. **Is it okay to leave varicocele untreated?**
   If it is not treated, since blood cannot be removed from the testes, it leads to deterioration in sperm quality, infertility, and complaints of pain.
5. **How to fix varicocele without surgery?**
   It can be treated without surgery by the embolization method using a coil and an embolic plug.

SECTION 2: PROCEDURE DETAILS

6. **Are you awake during embolization?**
   Yes, it is done painlessly using only four or five cc of local anesthetic.
7. **Is embolization painful?**
   No; the maximum pain that will be felt may be as much as the pain felt when blood is drawn from the arm.

8. **How long is varicocele embolization?**

   It takes about 30 or 45 minutes.

9. **How to prepare for embolization?**

   If the patient has no special condition, no additional preparation is necessary.

10. **Is embolization a major surgery?**

    No, it is not considered so; it is an extremely short and micro procedure.

SECTION 3: EFFECTIVENESS AND OUTCOMES

11. **How successful is varicocele embolization?**

    It is over 95%.

12. **Does sperm improve after varicocele embolization?**

    Since the insufficient veins are treated, sperm quality increases.

13. **Does testosterone increase after varicocele embolization?**

    Actually, this depends on a few factors; if there is testosterone deficiency due to varicocele disease, it increases.

14. **How long does varicocele embolization last?**

    A 5% recurrence has been reported both in surgical treatment and in embolization treatment; if there is no recurrence, it is effective for a lifetime.

15. **Does embolization improve fertility?**

    If the patient has a decrease in fertility due to varicocele disease, it increases.

SECTION 4: RISKS AND COMPLICATIONS

16. **Can complications occur during varicocele embolization??**

    Every procedure has complications, but the probability of serious complications in the embolization procedure is very, very low.

17. **Can varicocele embolization fail?**

    If the patient does not have a very unusual anatomy, the probability of failure is very low.

18. **What are the long-term side effects of embolization?**

    No significant long-term side effect has been reported in studies.

19. **Can varicocele return after embolization?**

    It has been reported that the disease recurs in about 5% of patients.

20. **Can embolization cause infertility?**

    No, it is used in the treatment of infertility.

SECTION 5: RECOVERY PROCESS

21. **How long is recovery from varicocele embolization?**

    An increase in sperm quality is expected within one to three months after treatment.

22. **Can I walk after varicocele embolization?**

    The patient can start walking immediately after the procedure.

23. **How to sleep after varicocele embolization?**

    As a precaution after the procedure, heavy lifting should be avoided for the first month and the patient should not strain too much.

24. **Can I do gym after varicocele embolization?**

    As a precaution, even if one goes to the gym in the first month, movements that increase intra-abdominal pressure should be avoided.

25. **What happens to veins after embolization?**

    The insufficient veins are occluded and, over time, undergo atrophy and shrink.
