# Supplementary material for: Comparison of artificial intelligence models and physicians in patient education for varicocele embolization: a double-blind randomized controlled trial
Source: Front Radiol. 2025 Oct 14;5:1682725. doi: 10.3389/fradi.2025.1682725 (PMC12558931; doi:10.3389/fradi.2025.1682725)
Supplement: Supplementary file 2 [file Datasheet2.pdf]

SORULAR VE CEVAPLARI: Yapay Zeka ve İnsan (Girişimsel Radyolog) tarafından varikosel embolizasyonu ile ilgili en sık sorulan 25 soruya verilen cevaplar.

CHAT GPT

BÖLÜM 1: GENEL BİLGİ VE TEDAVİ SEÇENEKLERİ

1. Varikosel için embolizasyon iyi bir tedavi midir?

Evet, embolizasyon minimal invaziv bir yöntemdir ve çoğu hasta için etkili bir tedavi seçeneğidir.

2. Varikosel embolizasyonunun avantajları ve dezavantajları nelerdir?

Avantajları: Ameliyatsız, hızlı iyileşme, lokal anestezi. Dezavantajları: Radyolojik ekipman ihtiyacı, nüks riski, her hastaya uygun olmama.

3. Hangi varikosel tedavisi en iyisidir: Mikrocerrahi mi, embolizasyon mu?

Mikrocerrahi daha düşük nüks ve daha yüksek başarı oranlarına sahiptir. Ancak embolizasyon daha az invazivdir. Hasta seçimine göre değişir.

4. Varikosel tedavi edilmezse ne olur?

İnfertilite, testis hacminde küçülme ve ağrı gibi sorunlara yol açabilir.

5. Varikoseli ameliyatsız tedavi etmenin yolları nelerdir?

Embolizasyon, yaşam tarzı değişiklikleri, destekleyici iç çamaşır kullanımı ve soğuk uygulamalar.

BÖLÜM 2: İŞLEM DETAYLARI

6. Embolizasyon işlemi sırasında uyanık mı olunur?

Evet, işlem sırasında hasta genellikle lokal anestezi altındadır ve uyanıktır.

7. Embolizasyon ağrılı bir işlem midir?

İşlem sırasında genellikle hafif rahatsızlık hissedilir, ciddi ağrı nadirdir.

8. Embolizasyon işlemi ne kadar sürer?

Genellikle 30 ila 60 dakika arasında sürer.

9. Embolizasyon için nasıl hazırlık yapılır?

Aç karnına gelinmesi, kan testlerinin yapılması ve alerji geçmişinin bildirilmesi gerekir.

10. Embolizasyon büyük bir ameliyat sayılır mı?

Hayır, embolizasyon minimal invaziv bir girişimdir, büyük ameliyat sınıfına girmez.

BÖLÜM 3: ETKİNLİK VE SONUÇLAR

11. Varikosel embolizasyonunun başarı oranı nedir?

%85–95 civarında teknik başarı oranına sahiptir.

12. Varikosel embolizasyonu sonrası sperm kalitesi artar mı?

Evet, çoğu erkekte sperm sayısı ve hareketliliğinde artış görülür.

13. Embolizasyon sonrası testosteron seviyesi artar mı?

Bazı hastalarda hafif bir artış olabilir, ancak her zaman belirgin değildir.

14. Varikosel embolizasyonu ne kadar süre etkilidir?

Yıllarca etkili olabilir, ancak nüks ihtimali %10-20 civarındadır.

15. Embolizasyon doğurganlığı artırır mı?

Evet, özellikle sperm parametreleri bozuk olan erkeklerde doğurganlık oranları artabilir.

BÖLÜM 4: RİSKLER VE KOMPLİKASYONLAR

16. Varikosel embolizasyonu sırasında işler ters gidebilir mi?

Nadir de olsa damar zedelenmesi, enfeksiyon veya başarısızlık riski vardır.

17. Varikosel embolizasyonu başarısız olabilir mi?

Evet, %5-10 oranında damar tam tıkanamayabilir veya tekrar açılabilir.

18. Varikosel embolizasyonunun uzun dönem yan etkileri nelerdir?

Uzun vadeli ciddi yan etkiler nadirdir; bazı hastalarda hafif kronik ağrı olabilir.

19. Varikosel, embolizasyon sonrası geri gelebilir mi?

Evet, %10-20 oranında nüks edebilir.

20. Embolizasyon kısırlığa neden olabilir mi?

Hayır, doğru yapıldığında kısırlık riski yaratmaz; genellikle doğurganlığı artırır.

BÖLÜM 5: İYİLEŞME SÜRECİ

21. Varikosel embolizasyonu sonrası iyileşme ne kadar sürer?

Çoğu hasta 1-2 gün içinde normal yaşantısına döner.

22. Embolizasyon sonrası ne zaman yürüyebilirim?

İşlemden birkaç saat sonra yürüyebilirsiniz.

23. Embolizasyon sonrası nasıl uyunmalıdır?

İlk birkaç gün sırt üstü veya hafif yükseltilmiş pozisyonda uyumak önerilir.

24. Embolizasyon sonrası spor salonuna ne zaman gidebilirim?

Genellikle 1 hafta sonra hafif egzersizlere başlanabilir, ağır sporlar için 2-3 hafta beklenir.

25. Embolizasyon sonrası damarlara ne olur?

Tedavi edilen damarlar kapanır ve zamanla vücut tarafından emilir veya küçülür.

GEMİNİ

BÖLÜM 1: GENEL BİLGİ VE TEDAVİ SEÇENEKLERİ

1. Varikosel için embolizasyon iyi bir tedavi midir?

Evet, embolizasyon varikosel için iyi ve minimal invaziv bir tedavi seçeneğidir. Ameliyata iyi bir alternatif olarak kabul edilir ve genellikle daha hızlı iyileşme süreci sunar.

2. Varikosel embolizasyonunun avantajları ve dezavantajları nelerdir?

Avantajları:

- Minimal İnvaziv: Büyük bir kesi yapılmaz, sadece küçük bir iğne deliği yeterlidir.
- Daha Hızlı İyileşme: Ameliyata göre iyileşme süresi genellikle daha kısadır.
- Ayakta Tedavi: Çoğu zaman hastanede yatış gerektirmez, aynı gün eve dönülebilir.
- Daha Az Ağrı: İşlem sonrası ağrı genellikle ameliyata göre daha hafiftir.
- Lokal Anestezi: Genellikle lokal anestezi altında yapılır, genel anestezi riskleri yoktur.
- Her İki Tarafa Uygulanabilirlik: Hem tek taraflı hem de çift taraflı varikosellerde uygulanabilir.

Dezavantajları:

- Tekrarlama Riski: Nadiren de olsa varikosel tekrarlayabilir.
- Radyasyon Maruziyeti: İşlem sırasında bir miktar radyasyon kullanılır.

- Kontrast Madde Reaksiyonu: Nadiren alerjik reaksiyonlar görülebilir.
- Kısıtlı Tecrübe: Her merkezde yeterli tecrübeye sahip uzman bulunmayabilir.

3. **Hangi varikosel tedavisi en iyisidir: Mikrocerrahi mi, embolizasyon mu?**

En iyi tedavi kişisel duruma bağlıdır. Hem mikrocerrahi hem de embolizasyon etkili tedavi yöntemleridir. Mikrocerrahi, nüks oranı açısından genellikle biraz daha düşük olabilirken, embolizasyon daha az invaziv olması ve daha hızlı iyileşme sunmasıyla öne çıkar. Hangi yöntemin sizin için daha uygun olduğuna doktorunuzla konuşarak karar vermelisiniz.

4. **Varikosel tedavi edilmezse ne olur?**

- Tedavi edilmeyen varikosel ilerleyebilir ve şu sorunlara yol açabilir:
- Kısırlık: Sperm kalitesini ve sayısını olumsuz etkileyebilir.
- Testis Atrofisi (Küçülmesi): Testis boyutunda küçülmeye neden olabilir.
- Ağrı: Kronik veya tekrarlayan testis ağrısı görülebilir.
- Testosteron Düşüklüğü: Hormon seviyelerinde düşüşe yol açabilir.

5. **Varikoseli ameliyatsız tedavi etmenin yolları nelerdir?**

Embolizasyon, varikoseli ameliyatsız tedavi etmenin ana yoludur. Bunun dışında, varikoselin kendisini doğrudan ortadan kaldıracak başka bir ameliyatsız yöntem yoktur. Bazı durumlarda ağrı yönetimi için semptomatik tedaviler veya yaşam tarzı değişiklikleri önerilebilir, ancak bunlar varikoseli tedavi etmez.

BÖLÜM 2: İŞLEM DETAYLARI

6. **Embolizasyon işlemi sırasında uyanık mı olunur?**

Evet, genellikle embolizasyon işlemi sırasında uyanık olursunuz. İşlem lokal anestezi altında yapılır, bu sayede işlem yapılan bölge uyuşur. İhtiyaç halinde hafif bir sedasyon (sakinleştirici) da verilebilir.

7. **Embolizasyon ağrılı bir işlem midir?**

Hayır, genellikle embolizasyon ağrılı bir işlem değildir. Lokal anestezi uygulandığı için iğnenin batırıldığı ilk an dışında ciddi bir ağrı hissedilmez. İşlem sırasında hafif bir basınç veya rahatsızlık hissedilebilir.

8. **Embolizasyon işlemi ne kadar sürer?**

Varikosel embolizasyonu genellikle 30 dakika ile 1 saat arasında sürer. Ancak bu süre, varikoselin karmaşıklığına ve hastanın durumuna göre değişiklik gösterebilir.

9. **Embolizasyon için nasıl hazırlık yapılır?**

- Embolizasyon için hazırlık genellikle şunları içerir:
- Açlık: İşlemden belirli bir süre önce (genellikle 6-8 saat) aç kalmanız istenir.
- İlaçlar: Kullandığınız tüm ilaçları doktorunuza bildirmeniz gerekir. Kan sulandırıcı ilaçların işlem öncesinde kesilmesi gerekebilir.
- Sıvı Alımı: İşlem öncesinde yeterli sıvı alımı önemlidir.
- Giyim: Rahat kıyafetler giymeniz önerilir.
- Testler: İşlem öncesinde kan testleri ve diğer görüntüleme testleri istenebilir.

10. **Embolizasyon büyük bir ameliyat sayılır mı?**

Hayır, embolizasyon büyük bir ameliyat sayılmaz. Minimal invaziv bir girişimdir ve geleneksel açık ameliyata göre çok daha az riskli ve daha hızlı iyileşme süreci olan bir yöntemdir. Genellikle "ameliyat" yerine "girişimsel radyolojik işlem" olarak adlandırılır.

BÖLÜM 3: ETKİNLİK VE SONUÇLAR

11. Varikosel embolizasyonunun başarı oranı nedir?

Varikosel embolizasyonunun başarı oranı oldukça yüksektir, genellikle %90-95 civarındadır. Bu oran, varikoselin tamamen kapanması ve semptomların düzelmesi açısından değerlendirilir.

12. Varikosel embolizasyonu sonrası sperm kalitesi artar mı?

Evet, varikosel embolizasyonu sonrası sperm kalitesinde artış görülebilir. Özellikle sperm sayısı, hareketliliği ve morfolojisi üzerinde olumlu etkileri olduğu bilimsel çalışmalarla gösterilmiştir. Ancak her vakada aynı derecede iyileşme görülmeyebilir.

13. Embolizasyon sonrası testosteron seviyesi artar mı?

Evet, bazı çalışmalarda embolizasyon sonrası testosteron seviyelerinde artış olduğu gözlemlenmiştir. Varikoselin testosteron üretimi üzerindeki olumsuz etkisinin ortadan kalkmasıyla hormon seviyeleri normale dönebilir veya iyileşebilir.

14. Varikosel embolizasyonu ne kadar süre etkilidir?

Varikosel embolizasyonu kalıcı bir etki sağlamayı amaçlar. Başarılı bir şekilde kapatılan varisli damarlar genellikle kapalı kalır. Ancak nadiren de olsa farklı damarlar aracılığıyla yeni varikosel oluşumu veya mevcut varikoselin tekrarlaması mümkündür.

15. Embolizasyon doğurganlığı artırır mı?

Evet, embolizasyon infertilite (kısırlık) sorunu yaşayan erkeklerde doğurganlığı artırmaya yardımcı olabilir. Varikosel, ısı artışı ve kan birikimi nedeniyle sperm üretimini bozarak kısırlığa yol açabilir. Embolizasyon bu olumsuz etkileri ortadan kaldırarak doğal yolla veya yardımcı üreme teknikleriyle gebelik şansını artırabilir.

BÖLÜM 4: RİSKLER VE KOMPLİKASYONLAR

16. Varikosel embolizasyonu sırasında işler ters gidebilir mi?

Her tıbbi işlemde olduğu gibi, embolizasyonda da nadiren komplikasyonlar yaşanabilir, ancak bunlar genellikle hafiftir. Ters gitme durumu, büyük bir sorun yerine küçük

komplikasyonları ifade eder. Bunlar arasında enjeksiyon yerinde morarma, hafif ağrı veya alerjik reaksiyonlar bulunabilir. Ciddi komplikasyonlar çok nadirdir.

17. Varikosel embolizasyonu başarısız olabilir mi?

Evet, nadiren de olsa varikosel embolizasyonu başarısız olabilir. Başarısızlık, damarların tam olarak kapatılamaması veya varikoselin kısa süre sonra tekrarlaması şeklinde ortaya çıkabilir. Bu durumda başka bir tedavi yöntemi veya tekrar embolizasyon düşünülebilir.

18. Varikosel embolizasyonunun uzun dönem yan etkileri nelerdir?

Varikosel embolizasyonunun bilinen ciddi uzun dönem yan etkileri çok nadirdir. En sık görülen uzun dönem endişe, varikoselin tekrarlama olasılığıdır. Diğer potansiyel, ancak nadir görülen etkiler arasında kronik ağrı veya testis hasarı sayılabilir, ancak bunlar istisnai durumlardır.

19. Varikosel, embolizasyon sonrası geri gelebilir mi?

Evet, nadiren de olsa varikosel embolizasyon sonrası geri gelebilir (tekrarlayabilir). Bu durum, genellikle embolizasyon sırasında tam olarak kapatılamayan damarlar veya zamanla yeni damarların genişlemesi nedeniyle ortaya çıkabilir. Tekrarlama oranı mikroameliyata göre biraz daha yüksek olabilir ancak yine de düşüktür.

20. Embolizasyon kısırlığa neden olabilir mi?

Hayır, varikosel embolizasyonu kısırlığa neden olmaz. Aksine, varikosel nedeniyle oluşan kısırlığı tedavi etmeyi ve doğurganlığı artırmay ı amaçlayan bir yöntemdir.

BÖLÜM 5: İYİLEŞME SÜRECİ

21. Varikosel embolizasyonu sonrası iyileşme ne kadar sürer?

Varikosel embolizasyonu sonrası iyileşme genellikle çok hızlıdır. Çoğu hasta işlemden birkaç saat sonra normal aktivitelerine dönebilir. Tam iyileşme ve rahatlama birkaç gün içinde tamamlanır. Ağır fiziksel aktivitelerden birkaç gün uzak durulması önerilir.

22. Embolizasyon sonrası ne zaman yürüyebilirim?

Embolizasyon sonrası genellikle hemen yürüyebilirsiniz. İşlemden sonra kısa bir dinlenme süresi (yaklaşık 1-2 saat) sonrası ayağa kalkıp yürümekte bir sakınca yoktur.

23. Embolizasyon sonrası nasıl uyunmalıdır?

Embolizasyon sonrası özel bir uyku pozisyonu gerekliliği yoktur. Kendinizi en rahat hissettiğiniz pozisyonda uyuyabilirsiniz. Genellikle işlem bölgesinde hafif bir hassasiyet olabileceğinden, bu bölgeye baskı yapmayacak şekilde yatmak tercih edilebilir.

24. Embolizasyon sonrası spor salonuna ne zaman gidebilirim?

Embolizasyon sonrası spor salonuna dönüş süresi genellikle birkaç gündür. Hafif egzersizlere 2-3 gün sonra başlanabilirken, ağır kaldırma veya yoğun spor aktiviteleri için 1 hafta kadar beklenmesi önerilir. Doktorunuz size özel tavsiyelerde bulunacaktır.

25. Embolizasyon sonrası damarlara ne olur?

Embolizasyon sonrası varikosel oluşturan varisli damarlar kalıcı olarak kapatılır ve zamanla büzüşerek küçülür. Bu damarlar artık kan taşımaz ve kan akışı sağlıklı damarlar aracılığıyla devam eder. Vücut bu damarları zamanla absorbe eder.

COPİLOT

BÖLÜM 1: GENEL BİLGİ VE TEDAVİ SEÇENEKLERİ

1. Varikosel için embolizasyon iyi bir tedavi midir?

Evet, embolizasyon, varikosel tedavisinde etkili ve güvenli bir ameliyatsız seçenektir. Özellikle ameliyata alternatif arayanlar için uygundur. Başarı oranı %85–90 civarındadır ve çoğu vakada semptomları azaltır, sperm kalitesini iyileştirir.

2. Varikosel embolizasyonunun avantajları ve dezavantajları nelerdir?

Avantajlar:

- Ameliyatsızdır: Sadece damardan girilir, kesi yoktur.
- Lokal anesteziyle yapılır, genellikle uyanık olursun.
- Hızlı iyileşme: 1–2 gün içinde normal hayata dönülebilir.
- Daha az komplikasyon riski: Enfeksiyon, testis hasarı gibi cerrahi riskler düşüktür.
- İki taraflı varikosel varsa, tek seansta ikisi de tedavi edilebilir.

Dezavantajlar:

- Her merkezde uygulanmaz; girişimsel radyoloji uzmanı gerekir.
- %10–15 oranında nüks görülebilir.
- Nadiren embolizasyon materyali yer değiştirebilir.
- Bazı kişilerde geçici ağrı veya rahatsızlık olabilir.

3. Hangi varikosel tedavisi en iyisidir: Mikrocerrahi mi, embolizasyon mu?

Her iki yöntem de etkilidir. Karşılaştırma:

Özellik	Mikrocerrahi	Embolizasyon
Anestezi	Genel/spinal	Lokal
İyileşme süresi	2–3 hafta	1–2 gün
Kesi	Var	Yok
Nüks oranı	%10 civarı	%10–15 civarı

| İki taraflı tedavi | Ayrı iki kesi gerekir | Tek seansta yapılabilir |

Sonuç: Embolizasyon daha konforlu; mikrocerrahi ise bazı vakalarda daha düşük nüks oranı sunabilir.

4. Varikosel tedavi edilmezse ne olur?

Tedavi edilmeyen varikosel:

- Sperm üretimini bozabilir
- Testis küçülmesine (atrofi) yol açabilir
- Testosteron düşüklüğü, cinsel isteksizlik, erektil disfonksiyon gibi sorunlara neden olabilir
- Kısırlık riski artar

5. Varikoseli ameliyatsız tedavi etmenin yolları nelerdir?

- Embolizasyon: En etkili ameliyatsız yöntem.
- Yaşam tarzı değişiklikleri: Egzersiz, dar kıyafetlerden kaçınma, sıcak duşlardan uzak durma.
- Antioksidan takviyeler: C vitamini, çinko, koenzim Q10.
- Bitkisel destekler: Sınırlı bilimsel kanıt olsa da bazıları dolaşımı destekleyebilir.

BÖLÜM 2: İŞLEM DETAYLARI

6. Embolizasyon işlemi sırasında uyanık mı olunur?

Evet, genellikle lokal anestezi ve hafif sedasyon uygulanır. Uyanık olursun ama rahatlatıcı ilaçlar sayesinde işlem sırasında ağrı hissetmezsin.

7. Embolizasyon ağrılı bir işlem midir?

Hayır, ağrılı değildir. Sadece giriş bölgesinde hafif baskı veya sıcaklık hissi olabilir. Sonrasında birkaç gün hafif kasık ağrısı olabilir ama genellikle basit ağrı kesicilerle kontrol altına alınır.

8. Embolizasyon işlemi ne kadar sürer?

İşlem süresi genellikle 30–60 dakika arasındadır. Aynı gün taburcu olabilirsin.

9. Embolizasyon için nasıl hazırlık yapılır?

- Kan sulandırıcı ilaçlar varsa doktorunla görüşmelisin.
- İşlemden önce 4–6 saat açlık gerekebilir.
- Rahat kıyafetler giyilmeli.
- Yanında seni eve götürecek biri olmalı.

10. Embolizasyon büyük bir ameliyat sayılır mı?

Hayır, minimal invaziv bir işlemdir. Kesi yoktur, hastanede yatış gerekmez. Genellikle günübirlik yapılır.

BÖLÜM 3: ETKİNLİK VE SONUÇLAR

11. Varikosel embolizasyonunun başarı oranı nedir?

Başarı oranı %85–90 civarındadır. Sperm parametrelerinde ve ağrıda belirgin iyileşme sağlar.

12. Varikosel embolizasyonu sonrası sperm kalitesi artar mı?

- Sperm sayısı, hareketliliği ve şekli iyileşebilir.

13. Embolizasyon sonrası testosteron seviyesi artar mı?

- Testosteron düzeyinde hafif artış görülebilir.
- Etki genellikle 3 ay içinde ortaya çıkar.

14. Varikosel embolizasyonu ne kadar süre etkilidir?

Başarılı bir embolizasyonun etkisi kalıcıdır. Ancak bazı vakalarda nüks olabilir ve tekrar tedavi gerekebilir.

15. Embolizasyon doğurganlığı artırır mı?

Evet, özellikle sperm parametreleri bozulmuş erkeklerde doğurganlıkta artış gözlenebilir. Bazı çiftlerde gebelik oranları yükselir.

BÖLÜM 4: RİSKLER VE KOMPLİKASYONLAR

16. Varikosel embolizasyonu sırasında işler ters gidebilir mi?

Nadir de olsa:

- Embolizasyon materyali yanlış yere gidebilir
- Enfeksiyon, alerji, kanama olabilir
- İşlem başarısız olabilir

17. Varikosel embolizasyonu başarısız olabilir mi?

Evet, %10–15 oranında nüks yaşanabilir.

18. Varikosel embolizasyonunun uzun dönem yan etkileri nelerdir?

- Uzun vadede ciddi yan etki nadirdir.
- Bazı kişilerde testiste hafif ağrı veya damar sertliği olabilir.

19. Varikosel, embolizasyon sonrası geri gelebilir mi?

Evet, bazı vakalarda yeniden oluşabilir.

20. Embolizasyon kısırlığa neden olabilir mi?

Genellikle hayır. Hedef doğurganlığı artırmaktır; kısırlık riski oldukça düşüktür.

BÖLÜM 5: İYİLEŞME SÜRECİ

21. Varikosel embolizasyonu sonrası iyileşme ne kadar sürer?

1–2 gün içinde normal hayata dönülür.

22. Embolizasyon sonrası ne zaman yürüyebilirim?

Genellikle aynı gün yürünebilir.

23. Embolizasyon sonrası nasıl uyunmalıdır?

Sırt üstü ve rahat pozisyonda uyunması önerilir.

24. Embolizasyon sonrası spor salonuna ne zaman gidebilirim?

Genellikle 1 hafta sonra spor yapılabilir.

25. Embolizasyon sonrası damarlara ne olur?

Embolize edilen damarlar kapanır ve kan akışı sağlıklı damarlara yönlendirilir. Bu, testisteki basıncı azaltır ve semptomları düzeltir.

İNSAN

BÖLÜM 1: GENEL BİLGİ VE TEDAVİ SEÇENEKLERİ

1. **Varikosel için embolizasyon iyi bir tedavi midir?**
Evet çünkü bu hastalıkta oradaki hastalıklı damarların kapatılması gerekmektedir
2. **Varikosel embolizasyonunun avantajları ve dezavantajları nelerdir?**
Avantajlari hastaneye yatış gerektirmeden yapılabilmesi yaklaşık yarım saat 45 dakikalık bir işlem olması ve komplikasyon riskinin çok düşük olması dezavantajları aslında pek bulunmamakla birlikte radyasyon ve kontrast madde kullanımı sayılabilir
3. **Hangi varikosel tedavisi en iyisidir: Mikrocerrahi mi, embolizasyon mu?**
Çalışmalarda iki işlemin birbirine üstünlüğü bulunmamakla birlikte ben bir Girişimsel radyolog olarak Varikoselim olsa embolizasyon tedavisi tercih ederdim çünkü cerrahi olmadan küçük bir iğne deliğinden yapılan tedaviyi tercih ederim
4. **Varikosel tedavi edilmezse ne olur?**
Eğer tedavi edilmezse kan testislerden uzaklaştırılamayacağı için sperm kalitesinde bozulma kısırlık ve ağrı şikayetlerine neden olur
5. **Varikoseli ameliyatsız tedavi etmenin yolları nelerdir?**

Ameliyatsız olarak Embolzasyon yöntemiyle Coil ve Embolizan tıkaç ile tedavi edilebilir

BÖLÜM 2: İŞLEM DETAYLARI

6. Embolizasyon işlemi sırasında uyanık mı olunur?
Evet sadece dört veya beş cc lokal anestetik kullanılarak ağrısı olarak yapılır
7. Embolizasyon ağrılı bir işlem midir?
Hayır hissedilecek maksimum ağrı koldan kan alırken hissedilen ağrı kadar olabilir
8. Embolizasyon işlemi ne kadar sürer?

Yaklaşık 30 veya 45 dakika sürer

9. Embolizasyon için nasıl hazırlık yapılır?

Eğer hastanın özel bir durumu yoksa ek bir hazırlığa gerek yoktur

10. Embolizasyon büyük bir ameliyat sayılır mı?

Hayır sayılmaz son derece kısa ve mikro bir işlemdir

BÖLÜM 3: ETKİNLİK VE SONUÇLAR

11. Varikosel embolizasyonunun başarı oranı nedir?
%95'in üzerindedir
12. Varikosel embolizasyonu sonrası sperm kalitesi artar mı?

Yetmezlik olan damarlar tedavi olduğu için sperm kalitesi artar

13. Embolizasyon sonrası testosteron seviyesi artar mı?

Aslında bu birkaç faktöre bağlıdır eğer Varikosel hastalığına bağlı testosteron düşüklüğü varsa artar

14. Varikosel embolizasyonu ne kadar süre etkilidir?

Hastaların hem cerrahi tedavide hem embolizasyon tedavisinde %5 oranında nüks bildirilmiştir eğer nüks olmazsa ömür boyu etkilidir

15. Embolizasyon doğurganlığı artırır mı?

Eğer hastanın varikosel hastalığına bağlı doğurganlık azalması varsa arttırır

BÖLÜM 4: RİSKLER VE KOMPLİKASYONLAR

16. Varikosel embolizasyonu sırasında işler ters gidebilir mi?
Her işlemin komplikasyonu vardır ancak embolizasyon işleminde ciddi komplikasyon ihtimali çok çok düşüktür
17. Varikosel embolizasyonu başarısız olabilir mi?

Eğer hastanın çok farklı bir anatomisi yoksa başarısız olma ihtimali çok düşüktür

18. Varikosel embolizasyonunun uzun dönem yan etkileri nelerdir?

Çalışmalarda belirgin bir uzun dönem yan etki bildirilmemiştir

19. Varikosel, embolizasyon sonrası geri gelebilir mi?

Hastaların %5 kadarında hastalığın nüksettiği bildirilmiştir

20. Embolizasyon kısırlığa neden olabilir mi?

Hayır kısırlık tedavisinde kullanılır

BÖLÜM 5: İYİLEŞME SÜRECİ

21. Varikosel embolizasyonu sonrası iyileşme ne kadar sürer?

Tedavi sonrası bir ila üç ay içerisinde sperm kalitesinde artış beklenmektedir

22. Embolizasyon sonrası ne zaman yürüyebilirim?

İşlem sonrası hasta hemen yürümeye başlayabilir

23. Embolizasyon sonrası nasıl uyunmalıdır?

İşlem sonrası ilk bir ay tedbir amaçlı fazla ağır kaldırmaktan kaçınılmalı ve hasta çok fazla ıkınmamalı

24. Embolizasyon sonrası spor salonuna ne zaman gidebilirim?

Tedbir amaçlı ilk bir ay spor salonuna gidilse bile batın içi basıncı arttırıcı hareketlerden uzak durulmalı

25. Embolizasyon sonrası damarlara ne olur?

Yetmezlik olan damarlar tıkanır ve zamanla atrofiye uğrar küçülür
