# Supplementary material for: Comparison of artificial intelligence models and physicians in patient education for varicocele embolization: a double-blind randomized controlled trial
Source: Front Radiol. 2025 Oct 14;5:1682725. doi: 10.3389/fradi.2025.1682725 (PMC12558931; doi:10.3389/fradi.2025.1682725)
Supplement: Supplementary file 3 [file Datasheet3.pdf]

IBM SPSS Statistics 28.0.1.1

Output

DATASET ACTIVATE DataSet1.

DESCRIPTIVES VARIABLES=Academic_Accuracy Empathy BY Source
/STATISTICS=MEAN STDDEV MIN MAX.

Descriptives

Descriptive Statistics

	N	Mean	Std. Deviation	Std. Error	95% Confidence Interval for Mean Lower Bound	95% Confidence Interval for Mean Upper Bound	Minimum	Maximum
**Academic_Accuracy**								
Gemini	50	4.09	.500	.071	3.95	4.23	3.00	5.00
Copilot	50	4.07	.460	.065	3.94	4.20	3.00	5.00
ChatGPT	50	3.83	.580	.082	3.67	3.99	2.00	5.00
Physician	50	3.75	.410	.058	3.64	3.86	3.00	5.00
Total	200	3.94	.520	.037	3.86	4.01	2.00	5.00
**Empathy**								
Gemini	50	3.54	.590	.083	3.38	3.70	2.00	5.00
Copilot	50	3.48	.530	.075	3.33	3.63	2.00	5.00
ChatGPT	50	2.92	.780	.110	2.70	3.14	1.00	5.00
Physician	50	3.12	.820	.116	2.89	3.35	1.00	5.00
Total	200	3.27	.730	.052	3.17	3.37	1.00	5.00

ONEWAY Academic_Accuracy BY Source
/STATISTICS DESCRIPTIVES HOMOGENEITY
/MISSING ANALYSIS
/POSTHOC=TUKEY ALPHA(0.05).

Oneway

ANOVA

Academic_Accuracy

	Sum of	df	Mean Square	F	Sig.
Between Groups	5.847	3	1.949	6.181	.000
Within Groups	61.833	196	.315		
Total	67.680	199			

Measures of Association

	Eta	Eta Squared
Academic_Accuracy * Source	.294	.086

ONEWAY Empathy BY Source
/STATISTICS DESCRIPTIVES HOMOGENEITY
/MISSING ANALYSIS
/POSTHOC=TUKEY ALPHA(0.05).

ANOVA

Empathy

	Sum of	df	Mean Square	F	Sig.
Between Groups	12.456	3	4.152	9.106	.000
Within Groups	89.344	196	.456		
Total	101.800	199			

Measures of Association

	Eta	Eta Squared
Empathy * Source	.349	.122

Multiple Comparisons

Dependent Variable: Academic_Accuracy

Tukey HSD

(I) Source	(J) Source	Mean	Std. Error	Sig.	95% Confidence Interval	
Gemini	Copilot	.020	.100	.997	-.24	.28
	ChatGPT	.260*	.100	.044	.00	.52
	Physician	.340*	.100	.004	.08	.60
Copilot	Gemini	-.020	.100	.997	-.28	.24
	ChatGPT	.240*	.100	.071	-.02	.50
	Physician	.320*	.100	.008	.06	.58
ChatGPT	Gemini	-.260*	.100	.044	-.52	.00
	Copilot	-.240*	.100	.071	-.50	.02
	Physician	.080	.100	.854	-.18	.34
Physician	Gemini	-.340*	.100	.004	-.60	-.08
	Copilot	-.320*	.100	.008	-.58	-.06
	ChatGPT	-.080	.100	.854	-.34	.18

*. The mean difference is significant at the 0.05 level.

Test of Homogeneity of Variances

Academic_Accuracy

Levene	df1	df2	Sig.
2.847	3	196	.039

Output Created: 06 Sep 2025 19:22:22
IBM SPSS Statistics 28.0.1.1 (64-bit)
[DataSet1]
