# Supplementary material for: Comparison of artificial intelligence models and physicians in patient education for varicocele embolization: a double-blind randomized controlled trial
Source: Front Radiol. 2025 Oct 14;5:1682725. doi: 10.3389/fradi.2025.1682725 (PMC12558931; doi:10.3389/fradi.2025.1682725)
Supplement: Supplementary file 4 [file Datasheet4.pdf]

VARİCOCELE EMBOLİZATİON ANSWER REVİEW FORM

Reviewer 1 Date: 03.08.2025

Review Instructions

This form contains 25 questions about varicocele embolization and 4 different answers (A, B, C, D) for each question. Please rate each answer on a scale of 1–5 in the following 2 categories:

Scoring Criteria:

Academic Accuracy Score (1–5):

- 5: Completely accurate, compliant with current guidelines, complete information
- 4: Largely accurate, minor omissions present
- 3: Partially accurate, some inaccuracies present
- 2: Mostly incorrect, significant omissions
- 1: Completely incorrect, contains dangerous information

Empathy Score (1–5):

- 5: Very empathetic, comforting and supportive language for the patient
- 4: Empathetic approach, expressions that show understanding of the patient
- 3: Neutral approach, moderate level of empathy
- 2: Little empathy, cold approach
- 1: Robotic, does not understand the patient, cold

SECTION 1: GENERAL INFORMATION AND TREATMENT OPTIONS

Question 1: Is embolization a good treatment for varicocele?

A) Yes, embolization is a good and minimally invasive treatment option for varicocele. It is considered a good alternative to surgery and usually offers a faster recovery process.

Academic Accuracy: ☐1 ☐2 ☐3 x4 ☐5

Empathy Score: ☐1 ☐2 ☐3 x4 ☐5

B) Yes, because in this disease the diseased veins there need to be closed.

Academic Accuracy: ☐1 ☐2 ☐3 x4 ☐5

Empathy Score: x1 ☐2 ☐3 ☐4 ☐5

C) Yes, embolization is a minimally invasive method and an effective treatment option for most patients.

Academic Accuracy: ☐1 ☐2 ☐3 x4 ☐5

Empathy Score: ☐1 ☐2 x3 ☐4 ☐5

D) Yes, embolization is an effective and safe non-surgical option in the treatment of varicocele. It is especially suitable for those seeking an alternative to surgery. The success rate is around 85–90%, and in most cases it reduces symptoms and improves sperm quality.

Academic Accuracy: ☐1 ☐2 ☐3 x4 ☐5

Empathy Score: ☐1 x2 ☐3 x4 ☐5

Question 2: What are the advantages and disadvantages of varicocele embolization?

A) **Advantages:**

• It is non-surgical: Access is only through a vein, there is no incision.
• Performed with local anesthesia; you are usually awake.
• Fast recovery: You can return to normal life within 1–2 days.
• Lower risk of complications: Surgical risks such as infection and testicular injury are low.
• If there is bilateral varicocele, both can be treated in a single session.

Disadvantages:

• Not performed in every center; an interventional radiology specialist is required.
• A recurrence rate of 10–15% may be seen.
• Embolization material may rarely migrate.
• Temporary pain or discomfort may occur in some people.

Academic Accuracy: ☐1 ☐2 ☐3 ☐4 x5

Empathy Score: ☐1 ☐2 x3 ☐4 ☐5

B) **Advantages:** Non-surgical, rapid recovery, local anesthesia. **Disadvantages:** Need for radiologic equipment, risk of recurrence, not suitable for every patient.

Academic Accuracy: ☐1 ☐2 x3 ☐4 ☐5

Empathy Score: x1 ☐2 x3 ☐4 ☐5

C) **Advantages** are that it can be performed without hospitalization, it is approximately a half-hour to 45-minute procedure, and the risk of complications is very low; **disadvantages,** although there are actually not many, can include radiation and contrast-agent use.

Academic Accuracy: ☐1 ☐2 x3 ☐4 ☐5

Empathy Score: ☐1 ☐2 ☐3 x4 ☐5

D) **Advantages:**

· Minimally Invasive: No large incision is made; only a small needle hole is sufficient.

· Faster Recovery: Compared to surgery, the recovery period is usually shorter.

· Outpatient Treatment: Most of the time hospitalization is not required; one can return home the same day.

· Less Pain: Post-procedure pain is usually milder than surgery.

· Local Anesthesia: It is usually performed under local anesthesia; there are no general anesthesia risks.

· Applicability to Both Sides: It can be applied in both unilateral and bilateral varicoceles.

Disadvantages:

· Risk of Recurrence: Even if rarely, varicocele may recur.

· Radiation Exposure: A certain amount of radiation is used during the procedure.

· Contrast Agent Reaction: Allergic reactions may rarely be seen.

· Limited Experience: A specialist with sufficient experience may not be available in every center.

Academic Accuracy: ☐1 ☐2 ☐3 ☐4 x5

Empathy Score: ☐1 ☐2 ☐3 x4 ☐5

Question 3: Which varicocele treatment is best: microsurgery or embolization?

A) Microsurgery has lower recurrence and higher success rates. However, embolization is less invasive. It varies according to patient selection.

Academic Accuracy: ☐1 ☐2 x3 ☐4 ☐5

Empathy Score: ☐1 ☐2 x3 ☐4 ☐5

B) Both methods are effective.

Academic Accuracy: ☐1 ☐2 ☐3 ☐4 x5

Empathy Score: ☐1 ☐2 ☐3 ☐4 x5

C) The best treatment depends on the individual situation. Both microsurgery and embolization are effective treatment methods. While microsurgery may be slightly lower in terms of recurrence rate, embolization stands out by being less invasive and offering faster recovery. You should decide which method is more suitable for you by talking with your doctor.

Academic Accuracy: ☐1 ☐2 ☐3 x4 ☐5

Empathy Score: ☐1 ☐2 ☐3 x4 ☐5

D) Although studies do not show a superiority of one procedure over the other, as an interventional radiologist, if I had a varicocele I would prefer embolization treatment, because I would prefer treatment performed through a small needle hole without surgery.

Academic Accuracy: ☐1 ☐2 x3 ☐4 ☐5

Empathy Score: ☐1 ☐2 ☐3 x4 ☐5

Question 4: What happens if varicocele is not treated?

A) If it is not treated, since blood cannot be removed from the testes, it leads to deterioration in sperm quality, infertility, and complaints of pain.

Academic Accuracy: ☐1 ☐2 ☐3 x4 ☐5

Empathy Score: ☐1 x2 ☐3 ☐4 ☐5

B) Untreated varicocele may progress and lead to the following problems:

· Infertility: It can adversely affect sperm quality and count.

· Testicular Atrophy (Shrinkage): It may cause a reduction in testicular size.

· Pain: Chronic or recurrent testicular pain may be seen.

· Low Testosterone: It may lead to a decrease in hormone levels.

Academic Accuracy: ☐1 ☐2 x3 ☐4 ☐5

Empathy Score: ☐1 ☐2 x3 ☐4 ☐5

C) **Untreated varicocele:**

• May impair sperm production

• May lead to testicular shrinkage (atrophy)

• May cause problems such as low testosterone, decreased sexual desire, erectile dysfunction

• The risk of infertility increases

Academic Accuracy: ☐1 ☐2 ☐3 x4 ☐5

Empathy Score: ☐1 x2 ☐3 ☐4 ☐5

D) It may lead to problems such as infertility, a decrease in testicular volume, and pain.

Academic Accuracy: ☐1 x2 ☐3 ☐4 ☐5

Empathy Score: x1 ☐2 ☐3 ☐4 ☐5

Question 5: What are the ways to treat varicocele without surgery?

A) Embolization is the main way to treat varicocele without surgery. Apart from this, there is no other non-surgical method that directly eliminates varicocele. In some cases, symptomatic treatments or lifestyle changes may be recommended for pain management, but these do not treat varicocele.

Academic Accuracy: ☐1 ☐2 ☐3 x4 ☐5

Empathy Score: ☐1 ☐2 x3 ☐4 ☐5

B) It can be treated without surgery by the embolization method using a coil and an embolic plug.

Academic Accuracy: ☐1 ☐2 x3 ☐4 ☐5

Empathy Score: ☐1 x2 ☐3 ☐4 ☐5

C) Embolization, lifestyle changes, use of supportive underwear, and cold applications.

Academic Accuracy: ☐1 x2 x3 ☐4 ☐5

Empathy Score: ☐1 x2 ☐3 ☐4 ☐5

D) **Embolization:** The most effective non-surgical method.

• **Lifestyle changes:** Exercise, avoiding tight clothing, staying away from hot showers.

• **Antioxidant supplements:** Vitamin C, zinc, coenzyme Q10.

• **Herbal supports:** Although scientific evidence is limited, some may support circulation.

Academic Accuracy: ☐1 ☐2 ☐3 x4 ☐5

Empathy Score: ☐1 ☐2 x3 ☐4 ☐5

SECTION 2: PROCEDURE DETAILS

Question 6: Are you awake during the embolization procedure?

A) Yes, local anesthesia and mild sedation are usually applied. You are awake, but thanks to relaxing medications you do not feel pain during the procedure.

Academic Accuracy: ☐1 ☐2 ☐3 x4 ☐5

Empathy Score: ☐1 ☐2 ☐3 x4 ☐5

B) Yes, you are usually awake during embolization. The procedure is performed under local anesthesia, so the area where the procedure is performed becomes numb. If needed, mild sedation (a sedative) may also be given.

Academic Accuracy: ☐1 ☐2 ☐3 x4 ☐5

Empathy Score: ☐1 ☐2 ☐3 x4 ☐5

C) Yes, it is done painlessly using only four or five cc of local anesthetic.

Academic Accuracy: ☐1 ☐2 x3 ☐4 ☐5

Empathy Score: ☐1 ☐2 x3 ☐4 ☐5

D) Yes, during the procedure the patient is usually under local anesthesia and is awake.

Academic Accuracy: ☐1 ☐2 ☐3 x4 ☐5

Empathy Score: ☐1 ☐2 ☐3 x4 ☐5

Question 7: Is embolization a painful procedure?

A) No; the maximum pain that will be felt may be as much as the pain felt when blood is

drawn from the arm.

Academic Accuracy: ☐1 ☐2 ☐3 x4 ☐5

Empathy Score: ☐1 ☐2 ☐3 ☐4 x5

B) No, it is not painful. Only a mild pressure or warmth may be felt at the entry site. Afterwards, mild groin pain for a few days may occur but is usually controlled with simple painkillers.

Academic Accuracy: ☐1 ☐2 ☐3 x4 ☐5

Empathy Score: ☐1 ☐2 ☐3 x4 ☐5

C) No, embolization is generally not a painful procedure. Since local anesthesia is applied, apart from the first moment when the needle is inserted, severe pain is not felt. A mild pressure or discomfort may be felt during the procedure.

Academic Accuracy: ☐1 ☐2 ☐3 x4 ☐5

Empathy Score: ☐1 ☐2 ☐3 x4 ☐5

D) Usually a mild discomfort is felt during the procedure; severe pain is rare.

Academic Accuracy: ☐1 ☐2 x3 x4 ☐5

Empathy Score: ☐1 ☐2 x3 ☐4 ☐5

Question 8: How long does the embolization procedure take?

A) It takes about 30 or 45 minutes.

Academic Accuracy: ☐1 ☐2 ☐3 x4 ☐5

Empathy Score: ☐1 x2 ☐3 ☐4 ☐5

B) It usually takes between 30 and 60 minutes.

Academic Accuracy: ☐1 ☐2 ☐3 x4 ☐5

Empathy Score: ☐1 x2 ☐3 ☐4 ☐5

C) Varicocele embolization generally takes between 30 minutes and 1 hour. However, this duration may vary depending on the complexity of the varicocele and the patient’s condition.

Academic Accuracy: ☐1 ☐2 ☐3 ☐4 x5

Empathy Score: ☐1 ☐2 ☐3 x4 ☐5

D) The procedure time is usually 30–60 minutes. You can be discharged the same day.

Academic Accuracy: ☐1 ☐2 ☐3 ☐4 x5

Empathy Score: ☐1 ☐2 ☐3 ☐4 x5

Question 9: How should one prepare for embolization?

A) Preparation for embolization generally includes:

· Fasting: You are asked to fast for a certain period (usually 6–8 hours) before the procedure.

· Medications: You must inform your doctor about all medications you use. Blood-thinning drugs may need to be stopped before the procedure.

· Fluid Intake: Adequate fluid intake is important before the procedure.

· Clothing: It is recommended to wear comfortable clothes.

· Tests: Blood tests and other imaging tests may be requested before the procedure.

Academic Accuracy: ☐1 ☐2 ☐3 x4 ☐5

Empathy Score: ☐1 ☐2 x3 ☐4 ☐5

B) – If there are blood-thinning medications, you should consult your doctor.

• Fasting for 4–6 hours may be required before the procedure.

• Comfortable clothes should be worn.

• Someone should be with you to take you home.

Academic Accuracy: ☐1 ☐2 x3 ☐4 ☐5

Empathy Score: ☐1 ☐2 ☐3 x4 ☐5

C) If the patient has no special condition, no additional preparation is necessary.

Academic Accuracy: ☐1 x2 ☐3 ☐4 ☐5

Empathy Score: ☐1 x2 ☐3 ☐4 ☐5

D) It is necessary to come fasting, to have blood tests performed, and to report allergy history.

Academic Accuracy: ☐1 ☐2 x3 ☐4 ☐5

Empathy Score: ☐1 x2 ☐3 ☐4 ☐5

Question 10: Is embolization considered a major operation?

A) No, embolization is a minimally invasive intervention; it does not fall into the major surgery class.

Academic Accuracy: ☐1 ☐2 ☐3 x4 ☐5

Empathy Score: ☐1 ☐2 x3 ☐4 ☐5

B) No, embolization is not considered a major operation. It is a minimally invasive intervention and, compared to traditional open surgery, it is much less risky and has a faster recovery process. It is generally referred to as an “interventional radiological procedure” rather than “surgery.”

Academic Accuracy: ☐1 ☐2 ☐3 x4 ☐5

Empathy Score: ☐1 ☐2 ☐3 x4 ☐5

C) No, it is a minimally invasive procedure. There is no incision, and hospitalization is not required. It is usually day-case.

Academic Accuracy: ☐1 ☐2 ☐3 x4 ☐5

Empathy Score: ☐1 ☐2 ☐3 x4 ☐5

D) No, it is not considered so; it is an extremely short and micro procedure.

Academic Accuracy: ☐1 ☐2 x3 ☐4 ☐5

Empathy Score: ☐1 ☐2 ☐3 x4 ☐5

SECTION 3: EFFECTIVENESS AND OUTCOMES

Question 11: What is the success rate of varicocele embolization?

A) The success rate is around 85–90%. It provides a marked improvement in sperm parameters and pain.

Academic Accuracy: ☐1 ☐2 ☐3 ☐4 x5

Empathy Score: ☐1 ☐2 ☐3 x4 ☐5

B) It is over 95%.

Academic Accuracy: ☐1 ☐2 ☐3 x4 ☐5

Empathy Score: ☐1 ☐2 x3 ☐4 ☐5

C) The success rate of varicocele embolization is quite high, usually around 90–95%. This rate is evaluated in terms of the varicocele being completely closed and the symptoms improving.

Academic Accuracy: ☐1 ☐2 ☐3 ☐4 x5

Empathy Score: ☐1 ☐2 ☐3 x4 ☐5

D) It has a technical success rate of around 85–95%.

Academic Accuracy: ☐1 ☐2 ☐3 x4 ☐5

Empathy Score: ☐1 ☐2 x3 ☐4 ☐5

Question 12: Does sperm quality increase after varicocele embolization?

A) Since the insufficient veins are treated, sperm quality increases.

Academic Accuracy: ☐1 ☐2 ☐3 x4 ☐5

Empathy Score: ☐1 ☐2 x3 ☐4 ☐5

B) Yes, in most men an increase in sperm count and motility is observed.

Academic Accuracy: ☐1 ☐2 ☐3 x4 ☐5

Empathy Score: ☐1 ☐2 x3 ☐4 ☐5

C) – Sperm count, motility, and morphology may improve.

Academic Accuracy: ☐1 ☐2 x3 ☐4 ☐5

Empathy Score: ☐1 x2 ☐3 ☐4 ☐5

D) Yes, an increase in sperm quality may be seen after varicocele embolization. Its positive effects on sperm count, motility, and morphology have been shown by scientific studies. However, the same degree of improvement may not be seen in every case.

Academic Accuracy: ☐1 ☐2 ☐3 x4 ☐5

Empathy Score: ☐1 ☐2 ☐3 x4 ☐5

Question 13: Does testosterone level increase after embolization?

A) Actually, this depends on a few factors; if there is testosterone deficiency due to varicocele disease, it increases.

Academic Accuracy: ☐1 ☐2 ☐3 x4 ☐5

Empathy Score: ☐1 ☐2 x3 ☐4 ☐5

B) – A mild increase in testosterone level may be seen.

• The effect usually appears within 3 months.

Academic Accuracy: ☐1 ☐2 x3 ☐4 ☐5

Empathy Score: ☐1 ☐2 x3 ☐4 ☐5

C) Yes, in some studies an increase in testosterone levels after embolization has been observed. With the elimination of the negative effect of varicocele on testosterone production, hormone levels may return to normal or improve.

Academic Accuracy: ☐1 ☐2 ☐3 ☐4 x5

Empathy Score: ☐1 ☐2 x3 ☐4 ☐5

D) There may be a slight increase in some patients, but it is not always evident.

Academic Accuracy: ☐1 ☐2 ☐3 ☐4 x5

Empathy Score: ☐1 x2 ☐3 ☐4 ☐5

Question 14: How long is varicocele embolization effective?

A) A recurrence rate of 5% has been reported in both surgical treatment and embolization treatment; if there is no recurrence, it is effective for a lifetime.

Academic Accuracy: ☐1 ☐2 ☐3 x4 ☐5

Empathy Score: ☐1 ☐2 ☐3 x4 ☐5

B) The effect of a successful embolization is permanent. However, recurrence may occur in some cases and re-treatment may be required.

Academic Accuracy: ☐1 ☐2 ☐3 x4 ☐5

Empathy Score: ☐1 ☐2 x3 ☐4 ☐5

C) Varicocele embolization aims to provide a permanent effect. The varicose veins that are successfully closed generally remain closed. However, even if rarely, the formation of a new varicocele through different veins or the recurrence of the existing varicocele is possible.

Academic Accuracy: ☐1 ☐2 ☐3 ☐4 x5

Empathy Score: ☐1 ☐2 ☐3 x4 ☐5

D) It can be effective for years, but the probability of recurrence is around 10–20%.

Academic Accuracy: ☐1 ☐2 ☐3 x4 ☐5

Empathy Score: ☐1 ☐2 ☐3 x4 ☐5

Question 15: Does embolization increase fertility?

A) Yes, especially in men with impaired sperm parameters, an increase in fertility may be observed. In some couples, pregnancy rates increase.

Academic Accuracy: ☐1 ☐2 ☐3 x4 ☐5

Empathy Score: ☐1 ☐2 ☐3 x4 ☐5

B) Yes, embolization can help increase fertility in men who have an infertility problem. Varicocele can lead to infertility by disrupting sperm production due to heat increase and blood pooling. Embolization eliminates these adverse effects and increases the chance of pregnancy by natural means or with assisted reproductive techniques.

Academic Accuracy: ☐1 ☐2 ☐3 ☐4 x5

Empathy Score: ☐1 ☐2 ☐3 x4 ☐5

C) If the patient has a decrease in fertility due to varicocele disease, it increases.

Academic Accuracy: ☐1 ☐2 ☐3 x4 ☐5

Empathy Score: ☐1 ☐2 x3 ☐4 ☐5

D) Yes, fertility rates may increase especially in men with impaired sperm parameters.

Academic Accuracy: ☐1 ☐2 ☐3 x4 ☐5

Empathy Score: ☐1 ☐2 x3 ☐4 ☐5

SECTION 4: RISKS AND COMPLICATIONS

Question 16: Can complications occur during varicocele embolization?

A) Every procedure has complications, but the probability of serious complications in the embolization procedure is very, very low.

Academic Accuracy: ☐1 ☐2 ☐3 x4 ☐5

Empathy Score: ☐1 ☐2 ☐3 x4 ☐5

B) Although rare, there is a risk of vessel injury, infection, or failure.

Academic Accuracy: ☐1 ☐2 ☐3 x4 ☐5

Empathy Score: ☐1 x2 ☐3 ☐4 ☐5

C) Although rare:

• The embolization material may go to the wrong place

• Infection, allergy, bleeding may occur

• The procedure may fail

Academic Accuracy: ☐1 ☐2 ☐3 ☐4 x5

Empathy Score: ☐1 ☐2 x3 ☐4 ☐5

D) As with any medical procedure, complications may rarely occur in embolization, but these are usually mild. "Going wrong" refers to minor complications rather than a major problem. These include bruising at the injection site, mild pain, or allergic reactions. Serious complications are very rare.

Academic Accuracy: ☐1 ☐2 ☐3 ☐4 x5

Empathy Score: ☐1 ☐2 ☐3 x4 ☐5

Question 17: Can varicocele embolization fail?

A) If the patient does not have a very unusual anatomy, the probability of failure is very low.

Academic Accuracy: ☐1 ☐2 ☐3 x4 ☐5

Empathy Score: ☐1 ☐2 ☐3 x4 ☐5

C) Yes, a recurrence rate of 10–15% may be experienced.

Academic Accuracy: ☐1 ☐2 ☐3 x4 ☐5

Empathy Score: ☐1 ☐2 x3 ☐4 ☐5

B) Yes, although rarely, varicocele embolization may fail. Failure may present as the veins not being completely occluded or the varicocele recurring shortly after. In this case, another treatment method or repeat embolization may be considered.

Academic Accuracy: ☐1 ☐2 ☐3 ☐4 x5

Empathy Score: ☐1 ☐2 ☐3 x4 ☐5

D) Yes, in 5–10% the vein may not be completely occluded or may reopen.

Academic Accuracy: ☐1 ☐2 ☐3 ☐4 x5

Empathy Score: ☐1 ☐2 ☐3 x4 ☐5

Question 18: What are the long-term side effects of varicocele embolization?

A) No significant long-term side effect has been reported in studies.

Academic Accuracy: ☐1 ☐2 ☐3 x4 ☐5

Empathy Score: ☐1 ☐2 ☐3 x4 ☐5

B) – Serious long-term side effects are rare.

• In some people there may be mild testicular pain or venous sclerosis.

Academic Accuracy: ☐1 ☐2 ☐3 x4 ☐5

Empathy Score: ☐1 ☐2 ☐3 x4 ☐5

C) Serious long-term side effects are rare; some patients may have mild chronic pain.

Academic Accuracy: ☐1 ☐2 ☐3 x4 ☐5

Empathy Score: ☐1 ☐2 ☐3 x4 ☐5

D) Known serious long-term side effects of varicocele embolization are very rare. The most common long-term concern is the possibility of recurrence of the varicocele. Other potential but rare effects include chronic pain or testicular injury, but these are exceptional.

Academic Accuracy: ☐1 ☐2 ☐3 ☐4 x5

Empathy Score: ☐1 ☐2 ☐3 x4 ☐5

Question 19: Can varicocele return after embolization?

A) It has been reported that the disease recurs in about 5% of patients.

Academic Accuracy: ☐1 ☐2 ☐3 ☐4 x5

Empathy Score: ☐1 ☐2 x3 ☐4 ☐5

B) Yes, it may recur at a rate of 10–20%.

Academic Accuracy: ☐1 x2 ☐3 ☐4 ☐5

Empathy Score: ☐1 x2 ☐3 ☐4 ☐5

C) Yes, it may reoccur in some cases.

Academic Accuracy: ☐1 x2 ☐3 ☐4 ☐5

Empathy Score: ☐1 x2 ☐3 ☐4 ☐5

D) Yes, although rarely, varicocele may return (recur) after embolization. This usually occurs due to veins that were not completely occluded during embolization or the dilation of new veins over time. The recurrence rate may be slightly higher than with microsurgery, but it is still low.

Academic Accuracy: ☐1 ☐2 ☐3 ☐4 x5

Empathy Score: ☐1 ☐2 ☐3 x4 ☐5

Question 20: Can embolization cause infertility?

A) No, it is used in the treatment of infertility.

Academic Accuracy: ☐1 ☐2 ☐3 x4 ☐5

Empathy Score: ☐1 ☐2 x3 ☐4 ☐5

B) No, varicocele embolization does not cause infertility. On the contrary, it is a method that aims to treat infertility caused by varicocele and to increase fertility.

Academic Accuracy: ☐1 ☐2 ☐3 x4 ☐5

Empathy Score: ☐1 ☐2 ☐3 x4 ☐5

C) Generally no. The goal is to increase fertility; the risk of infertility is quite low.

Academic Accuracy: ☐1 ☐2 ☐3 x4 ☐5

Empathy Score: ☐1 ☐2 ☐3 x4 ☐5

D) No, when done correctly it does not create a risk of infertility; it usually increases fertility.

Academic Accuracy: ☐1 ☐2 ☐3 x4 ☐5

Empathy Score: ☐1 ☐2 ☐3 x4 ☐5

SECTION 5: RECOVERY PROCESS

Question 21: How long does recovery take after varicocele embolization?

A) An increase in sperm quality is expected within one to three months after treatment.

Academic Accuracy: ☐1 ☐2 ☐3 x4 ☐5

Empathy Score: ☐1 ☐2 ☐3 x4 ☐5

B) Recovery after varicocele embolization is usually very fast. Most patients can return to normal activities a few hours after the procedure. Full recovery and relief are completed within a few days. It is recommended to stay away from heavy physical activities for a few days.

Academic Accuracy: ☐1 ☐2 ☐3 ☐4 x5

Empathy Score: ☐1 ☐2 ☐3 ☐4 x5

C) Most patients return to normal life within 1–2 days.

Academic Accuracy: ☐1 ☐2 ☐3 x4 ☐5

Empathy Score: ☐1 ☐2 ☐3 x4 ☐5

D) Return to normal life within 1–2 days.

Academic Accuracy: ☐1 ☐2 ☐3 x4 ☐5

Empathy Score: ☐1 ☐2 ☐3 x4 ☐5

Question 22: When can I walk after embolization?

A) The patient can start walking immediately after the procedure.

Academic Accuracy: ☐1 ☐2 ☐3 x4 ☐5

Empathy Score: ☐1 ☐2 ☐3 x4 ☐5

B) Usually you can walk the same day.

Academic Accuracy: ☐1 ☐2 ☐3 x4 ☐5

Empathy Score: ☐1 ☐2 ☐3 x4 ☐5

C) You can usually walk immediately after embolization. After a short rest period (about 1–2 hours) following the procedure, there is no harm in getting up and walking.

Academic Accuracy: ☐1 ☐2 ☐3 ☐4 x5

Empathy Score: ☐1 ☐2 ☐3 ☐4 x5

D) You can walk a few hours after the procedure.

Academic Accuracy: ☐1 ☐2 ☐3 ☐4 x5

Empathy Score: ☐1 ☐2 ☐3 x4 ☐5

Question 23: How should one sleep after embolization?

A) As a precaution after the procedure, heavy lifting should be avoided for the first month and the patient should not strain too much.

Academic Accuracy: ☐1 ☐2 ☐3 ☐4 x5

Empathy Score: ☐1 ☐2 ☐3 x4 ☐5

B) There is no requirement for a special sleep position after embolization. You can sleep in the position in which you feel most comfortable. Since there may be mild tenderness at the

procedure site, it is preferable to lie in a way that does not put pressure on this area.

Academic Accuracy: ☐1 ☐2 ☐3 ☐4 x5

Empathy Score: ☐1 ☐2 ☐3 ☐4 x5

C) It is recommended to sleep on your back or in a slightly elevated position for the first few days.

Academic Accuracy: ☐1 ☐2 ☐3 ☐4 x5

Empathy Score: ☐1 ☐2 x3 ☐4 ☐5

D) It is recommended to sleep on the back and in a comfortable position.

Academic Accuracy: ☐1 ☐2 ☐3 x4 ☐5

Empathy Score: ☐1 ☐2 x3 ☐4 ☐5

Question 24: When can I go to the gym after embolization?

A) As a precaution, even if one goes to the gym in the first month, movements that increase intra-abdominal pressure should be avoided.

Academic Accuracy: ☐1 ☐2 ☐3 ☐4 x5

Empathy Score: ☐1 ☐2 ☐3 x4 ☐5

B) The return time to the gym after embolization is usually a few days. While light exercises can be started 2–3 days later, it is recommended to wait about 1 week for heavy lifting or intense sports activities. Your doctor will give you personalized advice.

Academic Accuracy: ☐1 ☐2 ☐3 ☐4 x5

Empathy Score: ☐1 ☐2 ☐3 ☐4 x5

C) Usually light exercises can be started after 1 week; for heavy sports, 2–3 weeks are waited.

Academic Accuracy: ☐1 ☐2 ☐3 ☐4 x5

Empathy Score: ☐1 ☐2 ☐3 x4 ☐5

D) Usually you can exercise after 1 week.

Academic Accuracy: ☐1 ☐2 x3 ☐4 ☐5

Empathy Score: ☐1 ☐2 x3 ☐4 ☐5

Question 25: What happens to the veins after embolization?

A) The insufficient veins are occluded and over time undergo atrophy and shrink.

Academic Accuracy: ☐1 ☐2 ☐3 x4 ☐5

Empathy Score: ☐1 ☐2 ☐x3 ☐4 ☐5

B) After embolization, the varicose veins that cause varicocele are permanently closed and shrink by contracting over time. These veins no longer carry blood, and blood flow continues through healthy veins. The body absorbs these veins over time.

Academic Accuracy: ☐1 ☐2 ☐3 ☐4 x5

Empathy Score: ☐1 ☐2 ☐3 x4 ☐5

C) The treated veins close and, over time, are absorbed by the body or shrink.

Academic Accuracy: ☐1 ☐2 ☐3 x4 ☐5

Empathy Score: ☐1 ☐2 ☐3 x4 ☐5

D) The embolized veins close, and blood flow is directed to healthy veins. This reduces the pressure in the testis and corrects symptoms.

Academic Accuracy: ☐1 ☐2 ☐3 ☐4 x5

Empathy Score: ☐1 ☐2 ☐3 ☐4 x5
