# Supplementary material for: Comparison of artificial intelligence models and physicians in patient education for varicocele embolization: a double-blind randomized controlled trial
Source: Front Radiol. 2025 Oct 14;5:1682725. doi: 10.3389/fradi.2025.1682725 (PMC12558931; doi:10.3389/fradi.2025.1682725)
Supplement: Supplementary file 5 [file Datasheet5.pdf]

VARICOCELE EMBOLIZATION RESPONSE EVALUATION FORM

Reviewer 2 **Date:** 03/08/2025

Evaluation Instructions
This form contains 25 questions about varicocele embolization and four different answers (A, B, C, D) for each question. Please rate every answer on the two categories below using a 1–5 scale:

Scoring Criteria:
• **Academic Accuracy (1–5)**
 o **5:** Completely accurate, consistent with up-to-date guidelines, comprehensive information
 o **4:** Largely accurate, minor omissions present
 o **3:** Partly accurate, some errors present
 o **2:** Mostly inaccurate, important omissions exist
 o **1:** Completely inaccurate, contains dangerous information

• **Empathy (1–5)**
 o **5:** Very empathetic, reassuring and supportive language for the patient
 o **4:** Empathetic approach, statements showing understanding of the patient
 o **3:** Neutral approach, moderate empathy
 o **2:** Low empathy, cold approach
 o **1:** Robotic, does not understand the patient, cold

SECTION 1: GENERAL INFORMATION AND TREATMENT OPTIONS

Question 1: Is embolization a good treatment for varicocele?
A) Yes, embolization is a good and minimally invasive treatment option for varicocele. It is accepted as a good alternative to surgery and usually offers a faster recovery.
Academic Accuracy: ☐1 ☐2 ☐3 x4 ☐5
Empathy Score: ☐1 ☐2 ☐3 x4 ☐5

B) Yes, because in this disease the diseased veins there need to be closed.
Academic Accuracy: ☐1 ☐2 x3 ☐4 ☐5
Empathy Score: x1 ☐2 ☐3 ☐4 ☐5

C) Yes, embolization is a minimally invasive method and is an effective treatment option for most patients.
Academic Accuracy: ☐1 ☐2 ☐3 x4 ☐5
Empathy Score: ☐1 ☐2 x3 ☐4 ☐5

D) Yes, embolization is an effective and safe non-surgical option in the treatment of varicocele. It is especially suitable for those seeking an alternative to surgery. The success rate is about 85–90%, and in most cases it reduces symptoms and improves sperm quality.
Academic Accuracy: ☐1 ☐2 ☐3 ☐4 x5
Empathy Score: ☐1 ☐2 x3 ☐4 ☐5

Question 2: What are the advantages and disadvantages of varicocele embolization?
A) Advantages:
• It is non-surgical: only a single vein access is used, no incision.
• Performed under local anesthesia; you are usually awake.
• Fast recovery: you can return to normal life within 1–2 days.
• Lower complication risk: risks such as infection and testicular injury are lower than with surgery.
• If bilateral varicocele is present, both sides can be treated in a single session.
Disadvantages:
• Not performed in every center; requires an interventional radiologist.
• A recurrence rate of 10–15% may be seen.
• The embolic material may rarely migrate.
• Some people may experience temporary pain or discomfort.

Academic Accuracy: ☐1 ☐2 ☐3 ☐4 x5

Empathy Score: ☐1 ☐2 x3 ☐4 ☐5

B) Advantages: Non-surgical, fast recovery, local anesthesia. Disadvantages: Requires radiological equipment, risk of recurrence, not suitable for every patient.

Academic Accuracy: ☐1 ☐2 ☐3 x4 ☐5

Empathy Score: ☐1 x2 ☐3 ☐4 ☐5

C) Advantages include being performed without hospitalization, lasting about half an hour–45 minutes, and having a very low complication risk; disadvantages, while not many, may include radiation and contrast use.

Academic Accuracy: ☐1 ☐2 ☐3 x4 ☐5

Empathy Score: ☐1 ☐2 x3 ☐4 ☐5

D) Advantages:
· **Minimally Invasive:** No large incision; a small needle puncture suffices.
· **Faster Recovery:** Recovery is generally shorter than with surgery.
· **Outpatient Procedure:** Hospitalization is often unnecessary; you can go home the same day.
· **Less Pain:** Post-procedure pain is usually milder than after surgery.
· **Local Anesthesia:** Commonly performed under local anesthesia; avoids risks of general anesthesia.
· **Applicability to Both Sides:** Can be performed for unilateral or bilateral varicocele.
Disadvantages:
· **Risk of Recurrence:** Varicocele may recur, though rarely.
· **Radiation Exposure:** A certain amount of radiation is used during the procedure.
· **Contrast Reaction:** Allergic reactions are rare but possible.
· **Limited Experience:** Not every center has adequately experienced specialists.

Academic Accuracy: ☐1 ☐2 ☐3 ☐4 x5

Empathy Score: ☐1 ☐2 x3 ☐4 ☐5

Question 3: Which varicocele treatment is best: microsurgery or embolization?
A) Microsurgery has lower recurrence and higher success rates. However, embolization is less invasive. It depends on patient selection.
Academic Accuracy: ☐1 ☐2 x3 ☐4 ☐5
Empathy Score: ☐1 x2 ☐3 ☐4 ☐5

B) Both methods are effective.
Comparison:

Feature	Microsurgery	Embolization
Anesthesia	General/spinal	Local
Recovery time	2–3 weeks	1–2 days
Incision	Present	None
Recurrence rate	~10%	~10–15%
Bilateral treatment	Requires two separate incisions	Can be done in a single session

Conclusion: Embolization is more comfortable; microsurgery may offer a lower recurrence rate in some cases.
Academic Accuracy: ☐1 ☐2 ☐3 x4 ☐5
Empathy Score: ☐1 ☐2 x3 ☐4 ☐5

C) The best treatment depends on the individual case. Both microsurgery and embolization are effective treatment methods. Microsurgery may have a slightly lower recurrence rate, while embolization stands out for being less invasive with faster recovery. You should decide with your doctor which method is more suitable for you.
Academic Accuracy: ☐1 ☐2 x3 ☐4 ☐5
Empathy Score: ☐1 ☐2 ☐3 ☐4 x5

D) Although studies do not show superiority of one procedure over the other, as an interventional radiologist, if I had a varicocele, I would choose embolization because I would prefer a treatment performed through a small needle puncture without surgery.
Academic Accuracy: ☐1 ☐2 ☐3 x4 ☐5
Empathy Score: ☐1 ☐2 ☐3 ☐4 x5

Question 4: What happens if varicocele is not treated?
A) If left untreated, because blood cannot be removed from the testicles, it leads to deterioration in sperm quality, infertility, and pain complaints.
Academic Accuracy: ☐1 ☐2 x3 ☐4 ☐5
Empathy Score: ☐1 ☐2 x3 ☐4 ☐5

B) If untreated, varicocele may progress and cause the following problems:
· **Infertility:** It may adversely affect sperm quality and count.
· **Testicular Atrophy (Shrinkage):** May lead to a decrease in testicular size.
· **Pain:** Chronic or recurrent testicular pain can occur.
· **Low Testosterone:** May lead to decreased hormone levels.

Academic Accuracy: ☐1 ☐2 ☐3 ☐4 x5
Empathy Score: ☐1 ☐2 x3 ☐4 ☐5

C) Untreated varicocele:
• May impair sperm production
• May lead to testicular shrinkage (atrophy)
• May cause low testosterone, decreased libido, erectile dysfunction
• Increases risk of infertility
Academic Accuracy: ☐1 ☐2 ☐3 x4 ☐5
Empathy Score: ☐1 ☐2 x3 ☐4 ☐5

D) It may lead to problems such as infertility, decreased testicular volume, and pain.
Academic Accuracy: ☐1 ☐2 x3 ☐4 ☐5
Empathy Score: ☐1 ☐2 x3 ☐4 ☐5

Question 5: What are the non-surgical ways to treat varicocele?
A) Embolization is the main way to treat varicocele without surgery. Apart from this, there is no other non-surgical method that directly eliminates varicocele. In some cases, symptomatic treatments or lifestyle changes may be recommended for pain management, but these do not treat varicocele.
Academic Accuracy: ☐1 ☐2 ☐3 ☐4 x5
Empathy Score: ☐1 ☐2 ☐3 x4 ☐5

B) It can be treated without surgery by embolization using coils and vascular plugs.
Academic Accuracy: ☐1 x2 ☐3 ☐4 ☐5
Empathy Score: ☐1 x2 ☐3 ☐4 ☐5

C) Embolization, lifestyle changes, supportive underwear, and cold applications.
Academic Accuracy: ☐1 ☐2 ☐3 x4 ☐5
Empathy Score: ☐1 ☐2 x3 ☐4 ☐5

D) Embolization: The most effective non-surgical method.
• **Lifestyle changes:** Exercise, avoiding tight clothing, avoiding hot showers.
• **Antioxidant supplements:** Vitamin C, zinc, coenzyme Q10.
• **Herbal supports:** Although scientific evidence is limited, some may support circulation.
Academic Accuracy: ☐1 ☐2 ☐3 x4 ☐5
Empathy Score: ☐1 ☐2 x3 ☐4 ☐5

SECTION 2: PROCEDURE DETAILS

Question 6: Are you awake during the embolization procedure?
A) Yes, local anesthesia and light sedation are usually administered. You are awake, but thanks to relaxing medications you do not feel pain during the procedure.

Academic Accuracy: ☐1 ☐2 ☐3 ☐4 x5
Empathy Score: ☐1 ☐2 ☐3 ☐4 x5

B) Yes, you are generally awake during embolization. The procedure is performed under local anesthesia, so the area of the procedure becomes numb. If necessary, light sedation (a tranquilizer) may also be given.
Academic Accuracy: ☐1 ☐2 ☐3 ☐4 x5
Empathy Score: ☐1 ☐2 ☐3 ☐4 x5

C) Yes, it is performed painlessly using only four or five cc of local anesthetic.
Academic Accuracy: ☐1 ☐2 ☐3 xx4 ☐5
Empathy Score: ☐1 ☐2 x3 ☐4 ☐5

D) Yes, the patient is usually under local anesthesia and awake during the procedure.
Academic Accuracy: ☐1 ☐2 ☐3 x4 ☐5
Empathy Score: ☐1 ☐2 x3 ☐4 ☐5

Question 7: Is embolization a painful procedure?
A) No; the maximum pain felt may be about as much as when having blood drawn from the arm.
Academic Accuracy: ☐1 ☐2 x3 ☐4 ☐5
Empathy Score: ☐1 ☐2 x3 ☐4 ☐5

B) No, it is not painful. You may only feel mild pressure or warmth at the entry site. Afterwards, mild groin pain may last a few days but is usually controlled with simple painkillers.
Academic Accuracy: ☐1 ☐2 ☐3 ☐4 x5
Empathy Score: ☐1 ☐2 ☐3 x4 ☐5

C) No, embolization is generally not a painful procedure. Since local anesthesia is applied, apart from the initial needle prick, severe pain is not felt. Mild pressure or discomfort may be felt during the procedure.
Academic Accuracy: ☐1 ☐2 ☐3 ☐4 x5
Empathy Score: ☐1 ☐2 ☐3 x4 ☐5

D) Mild discomfort is usually felt during the procedure; severe pain is rare.
Academic Accuracy: ☐1 ☐2 ☐3 x4 ☐5
Empathy Score: ☐1 ☐2 ☐3 x4 ☐5

Question 8: How long does the embolization procedure take?
A) About 30 or 45 minutes.
Academic Accuracy: ☐1 ☐2 ☐3 x4 ☐5
Empathy Score: ☐1 ☐2 x3 ☐4 ☐5

B) Generally between 30 and 60 minutes.
Academic Accuracy: ☐1 ☐2 ☐3 x4 ☐5
Empathy Score: ☐1 ☐2 x3 ☐4 ☐5

C) Varicocele embolization usually takes 30 minutes to 1 hour. However, this duration may vary depending on the complexity of the varicocele and the patient's condition.
Academic Accuracy: ☐1 ☐2 ☐3 ☐4 x5
Empathy Score: ☐1 ☐2 x3 ☐4 ☐5

D) The procedure duration is generally 30–60 minutes. You can be discharged the same day.
Academic Accuracy: ☐1 ☐2 ☐3 x4 ☐5
Empathy Score: ☐1 ☐2 ☐3 x4 ☐5

Question 9: How should one prepare for embolization?
A) Preparation for embolization generally includes the following:
· **Fasting:** You will be asked to fast for a certain period (usually 6–8 hours) before the procedure.
· **Medications:** You must inform your doctor of all medications you use. Blood thinners may need to be stopped before the procedure.
· **Fluid Intake:** Adequate fluid intake before the procedure is important.
· **Clothing:** Comfortable clothing is recommended.
· **Tests:** Blood tests and other imaging tests may be requested before the procedure.
Academic Accuracy: ☐1 ☐2 ☐3 ☐4 x5
Empathy Score: ☐1 ☐2 x3 ☐4 ☐5

B) – If you are on blood thinners, you should consult your doctor.
• Fasting 4–6 hours before the procedure may be required.
• Wear comfortable clothing.
• Have someone with you to take you home.
Academic Accuracy: ☐1 ☐2 ☐3 x4 ☐5
Empathy Score: ☐1 ☐2 x3 ☐4 ☐5

C) If the patient has no special condition, no additional preparation is needed.
Academic Accuracy: ☐1 ☐2 x3 ☐4 ☐5
Empathy Score: ☐1 x2 ☐3 ☐4 ☐5

D) You should come fasting, have blood tests done, and report any allergy history.
Academic Accuracy: ☐1 ☐2 ☐3 x4 ☐5
Empathy Score: ☐1 ☐2 x3 ☐4 ☐5

Question 10: Is embolization considered major surgery?
A) No, embolization is a minimally invasive procedure; it does not fall into the major surgery category.

Academic Accuracy: ☐1 ☐2 ☐3 x4 ☐5
Empathy Score: ☐1 x2 ☐3 ☐4 ☐5

B) No, embolization is not considered major surgery. It is a minimally invasive procedure and has much lower risk and faster recovery compared to traditional open surgery. It is generally referred to as an "interventional radiology procedure" rather than "surgery."
Academic Accuracy: ☐1 ☐2 ☐3 ☐4 x5
Empathy Score: ☐1 ☐2 ☐3 x4 ☐5

C) No, it is a minimally invasive procedure. There is no incision and hospitalization is not required. It is usually done as day surgery.
Academic Accuracy: ☐1 ☐2 ☐3 x4 ☐5
Empathy Score: ☐1 ☐2 x3 ☐4 ☐5

D) No, it is not considered so; it is an extremely short and micro procedure.
Academic Accuracy: ☐1 ☐2 ☐3 x4 ☐5
Empathy Score: ☐1 ☐2 x3 ☐4 ☐5

SECTION 3: EFFECTIVENESS AND OUTCOMES

Question 11: What is the success rate of varicocele embolization?
A) The success rate is about 85–90%. It provides marked improvement in sperm parameters and pain.
Academic Accuracy: ☐1 ☐2 ☐3 x4 ☐5
Empathy Score: ☐1 ☐2 x3 ☐4 ☐5

B) Over 95%.
Academic Accuracy: ☐1 ☐2 ☐3 x4 ☐5
Empathy Score: ☐1 ☐2 x3 ☐4 ☐5

C) The success rate of varicocele embolization is quite high, generally around 90–95%. This rate is assessed in terms of complete occlusion of varicose veins and improvement of symptoms.
Academic Accuracy: ☐1 ☐2 ☐3 x4 ☐5
Empathy Score: ☐1 ☐2 ☐3 x4 ☐5

D) The technical success rate is approximately 85–95%.
Academic Accuracy: ☐1 ☐2 ☐3 x4 ☐5
Empathy Score: ☐1 ☐2 x3 ☐4 ☐5

Question 12: Does sperm quality improve after varicocele embolization?
A) Since the insufficient veins are treated, sperm quality increases.

Academic Accuracy: ☐1 ☐2 x3 ☐4 ☐5
Empathy Score: ☐1 ☐2 ☐3 x4 ☐5

B) Yes, in most men there is an increase in sperm count and motility.
Academic Accuracy: ☐1 ☐2 ☐3 x4 ☐5
Empathy Score: ☐1 ☐2 x3 ☐4 ☐5

C) – Sperm count, motility, and morphology may improve.
Academic Accuracy: ☐1 ☐2 ☐3 x4 ☐5
Empathy Score: ☐1 ☐2 x3 ☐4 ☐5

D) Yes, an increase in sperm quality may be seen after varicocele embolization. Its positive effects on sperm count, motility, and morphology have been demonstrated by scientific studies. However, the degree of improvement may not be the same in every case.
Academic Accuracy: ☐1 ☐2 ☐3 ☐4 x5
Empathy Score: ☐1 ☐2 ☐3 x4 ☐5

Question 13: Does testosterone level increase after embolization?
A) In fact, this depends on several factors; if there is low testosterone due to varicocele, it increases.
Academic Accuracy: ☐1 ☐2 ☐3 x4 ☐5
Empathy Score: ☐1 ☐2 x3 ☐4 ☐5

B) – A slight increase in testosterone level may be observed.
• The effect usually appears within 3 months.
Academic Accuracy: ☐1 ☐2 ☐3 x4 ☐5
Empathy Score: ☐1 ☐2 x3 ☐4 ☐5

C) Yes, some studies have observed increases in testosterone levels after embolization. With the elimination of the adverse effect of varicocele on testosterone production, hormone levels may normalize or improve.
Academic Accuracy: ☐1 ☐2 ☐3 ☐4 x5
Empathy Score: ☐1 ☐2 ☐3 x4 ☐5

D) There may be a slight increase in some patients, but it is not always pronounced.
Academic Accuracy: ☐1 ☐2 ☐3 x4 ☐5
Empathy Score: ☐1 ☐2 x3 ☐4 ☐5

Question 14: How long is varicocele embolization effective?
A) A 5% recurrence rate has been reported for both surgical treatment and embolization; if there is no recurrence, it is effective for life.
Academic Accuracy: ☐1 ☐2 ☐3 ☐4 x5
Empathy Score: ☐1 ☐2 ☐3 x4 ☐5

B) The effect of a successful embolization is permanent. However, recurrence may occur in some cases and re-treatment may be required.
Academic Accuracy: ☐1 ☐2 ☐3 x4 ☐5
Empathy Score: ☐1 ☐2 x3 ☐4 ☐5

C) Varicocele embolization aims to provide a permanent effect. Varicose veins that are successfully occluded generally remain closed. However, rarely, new varicocele formation via different veins or recurrence of the existing varicocele is possible.
Academic Accuracy: ☐1 ☐2 ☐3 x4 ☐5
Empathy Score: ☐1 ☐2 x3 ☐4 ☐5

D) It may be effective for years, but the probability of recurrence is about 10–20%.
Academic Accuracy: ☐1 ☐2 ☐3 x4 ☐5
Empathy Score: ☐1 ☐2 x3 ☐4 ☐5

Question 15: Does embolization increase fertility?
A) Yes, especially in men with impaired sperm parameters, an increase in fertility can be seen. Pregnancy rates increase in some couples.
Academic Accuracy: ☐1 ☐2 ☐3 x4 ☐5
Empathy Score: ☐1 ☐2 x3 ☐4 ☐5

B) Yes, it can help increase fertility in men with infertility problems. Varicocele can cause infertility by disrupting sperm production due to increased heat and blood pooling. Embolization eliminates these adverse effects and increases the chance of pregnancy either naturally or with assisted reproductive techniques.
Academic Accuracy: ☐1 ☐2 ☐3 ☐4 x5
Empathy Score: ☐1 ☐2 x3 ☐4 ☐5

C) If the patient has reduced fertility due to varicocele, it increases.
Academic Accuracy: ☐1 ☐2 ☐3 x4 ☐5
Empathy Score: ☐1 ☐2 x3 ☐4 ☐5

D) Yes, fertility rates may increase especially in men with impaired sperm parameters.
Academic Accuracy: ☐1 ☐2 ☐3 x4 ☐5
Empathy Score: ☐1 ☐2 x3 ☐4 ☐5

SECTION 4: RISKS AND COMPLICATIONS

Question 16: Can things go wrong during varicocele embolization?
A) Every procedure has complications, but the likelihood of serious complications in embolization is very, very low.
Academic Accuracy: ☐1 ☐2 ☐3 x4 ☐5
Empathy Score: ☐1 ☐2 ☐3 x4 ☐5

B) Although rare, there is a risk of vessel injury, infection, or failure.
Academic Accuracy: ☐1 ☐2 ☐3 x4 ☐5
Empathy Score: ☐1 ☐2 x3 ☐4 ☐5

C) Rarely:
• The embolic material may go to an unintended place
• Infection, allergy, bleeding may occur
• The procedure may fail
Academic Accuracy: ☐1 ☐2 ☐3 ☐4 x5
Empathy Score: ☐1 ☐2 x3 ☐4 ☐5

D) As with any medical procedure, complications can rarely occur in embolization, but they are usually mild. "Things going wrong" refers to minor complications rather than a major problem. These include bruising at the injection site, mild pain, or allergic reactions. Serious complications are very rare.
Academic Accuracy: ☐1 ☐2 ☐3 ☐4 x5
Empathy Score: ☐1 ☐2 ☐3 x4 ☐5

Question 17: Can varicocele embolization fail?
A) Unless the patient has a very unusual anatomy, the chance of failure is very low.
Academic Accuracy: ☐1 ☐2 ☐3 x4 ☐5
Empathy Score: ☐1 ☐2 x3 ☐4 ☐5

B) Yes, a recurrence rate of 10–15% may be experienced.
Academic Accuracy: ☐1 ☐2 x3 ☐4 ☐5
Empathy Score: ☐1 x2 ☐3 ☐4 ☐5

C) Yes, varicocele embolization can rarely fail. Failure may present as inability to completely occlude the veins or recurrence of the varicocele shortly thereafter. In such a case, another treatment method or repeat embolization may be considered.
Academic Accuracy: ☐1 ☐2 ☐3 ☐4 x5
Empathy Score: ☐1 ☐2 ☐3 x4 ☐5

D) Yes, in 5–10% the vein may not be completely occluded or may reopen.
Academic Accuracy: ☐1 ☐2 ☐3 x4 ☐5
Empathy Score: ☐1 ☐2 X3 ☐4 ☐5

Question 18: What are the long-term side effects of varicocele embolization?
A) No significant long-term side effects have been reported in studies.
Academic Accuracy: ☐1 ☐2 ☐3 X4 ☐5
Empathy Score: ☐1 ☐2 X3 ☐4 ☐5

B) – Serious long-term side effects are rare.
• Some people may experience mild testicular pain or venous sclerosis.

Academic Accuracy: ☐1 X2 ☐3 ☐4 ☐5
Empathy Score: ☐1 ☐2 X3 ☐4 ☐5

C) Serious long-term side effects are rare; some patients may have mild chronic pain.
Academic Accuracy: ☐1 ☐2 ☐3 X4 ☐5
Empathy Score: ☐1 ☐2 X3 ☐4 ☐5

D) Known serious long-term side effects of varicocele embolization are very rare. The most common long-term concern is the possibility of recurrence of varicocele. Other potential but rare effects include chronic pain or testicular injury; however, these are exceptional.
Academic Accuracy: ☐1 ☐2 ☐3 ☐4 X5
Empathy Score: ☐1 ☐2 ☐3 X4 ☐5

Question 19: Can varicocele come back after embolization?
A) Recurrence of the disease has been reported in about 5% of patients.
Academic Accuracy: ☐1 ☐2 ☐3 ☐4 x5
Empathy Score: ☐1 ☐2 x3 ☐4 ☐5

B) Yes, it can recur at a rate of 10–20%.
Academic Accuracy: ☐1 x2 ☐3 ☐4 ☐5
Empathy Score: ☐1 ☐2 x3 ☐4 ☐5

C) Yes, it can recur in some cases.
Academic Accuracy: ☐1 ☐2 x3 ☐4 ☐5
Empathy Score: ☐1 x2 ☐3 ☐4 ☐5

D) Yes, although rare, varicocele may return (recur) after embolization. This usually occurs due to incomplete occlusion of the veins during embolization or dilation of new veins over time. The recurrence rate may be slightly higher than with microsurgery, but is still low.
Academic Accuracy: ☐1 ☐2 ☐3 x4 ☐5
Empathy Score: ☐1 ☐2 ☐3 x4 ☐5

Question 20: Can embolization cause infertility?
A) No, it is used in the treatment of infertility.
Academic Accuracy: ☐1 ☐2 x3 ☐4 ☐5
Empathy Score: ☐1 x2 ☐3 ☐4 ☐5

B) No, varicocele embolization does not cause infertility. On the contrary, it is a method that aims to treat infertility caused by varicocele and to increase fertility.
Academic Accuracy: ☐1 ☐2 ☐3 x4 ☐5
Empathy Score: ☐1 ☐2 ☐3 x4 ☐5

C) Generally no. The aim is to increase fertility; the risk of infertility is quite low.
Academic Accuracy: ☐1 ☐2 x3 ☐4 ☐5
Empathy Score: ☐1 ☐2 ☐3 x4 ☐5

D) No, when performed correctly it does not pose a risk of infertility; it usually increases fertility.
Academic Accuracy: ☐1 ☐2 ☐3 x4 ☐5
Empathy Score: ☐1 ☐2 ☐3 x4 ☐5

SECTION 5: RECOVERY PROCESS

Question 21: How long does recovery take after varicocele embolization?
A) An increase in sperm quality is expected one to three months after treatment.
Academic Accuracy: ☐1 ☐2 x3 ☐4 ☐5
Empathy Score: ☐1 ☐2 x3 ☐4 ☐5

B) Recovery after varicocele embolization is usually very fast. Most patients can return to normal activities a few hours after the procedure. Full recovery and relief are completed within a few days. It is recommended to avoid heavy physical activities for a few days.
Academic Accuracy: ☐1 ☐2 ☐3 ☐4 x5
Empathy Score: ☐1 ☐2 ☐3 x4 ☐5

C) Most patients return to normal life within 1–2 days.
Academic Accuracy: ☐1 ☐2 ☐3 x4 ☐5
Empathy Score: ☐1 ☐2 ☐3 x4 ☐5

D) Return to normal life within 1–2 days.
Academic Accuracy: ☐1 ☐2 ☐3 x4 ☐5
Empathy Score: ☐1 ☐2 x3 ☐4 ☐5

Question 22: When can I walk after embolization?
A) The patient can start walking immediately after the procedure.
Academic Accuracy: ☐1 ☐2 ☐3 x4 ☐5
Empathy Score: ☐1 ☐2 x3 ☐4 ☐5

B) You can usually walk on the same day.
Academic Accuracy: ☐1 ☐2 ☐3 x4 ☐5
Empathy Score: ☐1 ☐2 ☐3 x4 ☐5

C) You can generally walk immediately after embolization. There is no harm in getting up and walking after a short rest period (about 1–2 hours) following the procedure.
Academic Accuracy: ☐1 ☐2 ☐3 ☐4 x5
Empathy Score: ☐1 ☐2 ☐3 ☐4 x5

D) You can walk a few hours after the procedure.
Academic Accuracy: ☐1 ☐2 ☐3 x4 ☐5
Empathy Score: ☐1 ☐2 ☐3 x4 ☐5

Question 23: How should I sleep after embolization?
A) As a precaution during the first month after the procedure, heavy lifting should be avoided and the patient should avoid straining excessively.
Academic Accuracy: ☐1 ☐2 x3 ☐4 ☐5
Empathy Score: ☐1 ☐2 x3 ☐4 ☐5

B) There is no specific requirement for a sleep position after embolization. You can sleep in the position you feel most comfortable. Since there may be mild tenderness at the procedure site, it is preferable to lie in a way that does not put pressure on this area.
Academic Accuracy: ☐1 ☐2 ☐3 ☐4 x5
Empathy Score: ☐1 ☐2 ☐3 ☐4 x5

C) It is recommended to sleep on your back or in a slightly elevated position for the first few days.
Academic Accuracy: ☐1 ☐2 x3 ☐4 ☐5
Empathy Score: ☐1 ☐2 ☐3 x4 ☐5

D) It is recommended to sleep on your back and in a comfortable position.
Academic Accuracy: ☐1 ☐2 x3 ☐4 ☐5
Empathy Score: ☐1 ☐2 ☐3 x4 ☐5

Question 24: When can I go to the gym after embolization?
A) As a precaution, even if you go to the gym in the first month, you should avoid movements that increase intra-abdominal pressure.
Academic Accuracy: ☐1 ☐2 x3 ☐4 ☐5
Empathy Score: ☐1 ☐2 x3 ☐4 ☐5

B) The return to the gym after embolization is usually a few days. Light exercises can be started 2–3 days later; for heavy lifting or intense sports activities, it is recommended to wait about 1 week. Your doctor will provide personalized advice.
Academic Accuracy: ☐1 ☐2 ☐3 ☐4 x5
Empathy Score: ☐1 ☐2 ☐3 x4 ☐5

C) Light exercises can generally be started after 1 week; for heavy sports, wait 2–3 weeks.
Academic Accuracy: ☐1 ☐2 ☐3 ☐4 x5
Empathy Score: ☐1 ☐2 x3 ☐4 ☐5

D) You can generally exercise after 1 week.
Academic Accuracy: ☐1 ☐2 ☐3 x4 ☐5
Empathy Score: ☐1 ☐2 x3 ☐4 ☐5

Question 25: What happens to the veins after embolization?
A) The insufficient veins are occluded and over time atrophy and shrink.
Academic Accuracy: ☐1 ☐2 ☐3 x4 ☐5
Empathy Score: ☐1 ☐2 x3 ☐4 ☐5

B) After embolization, the varicose veins that cause varicocele are permanently closed and gradually contract and shrink. These veins no longer carry blood, and blood flow continues through healthy veins. The body gradually absorbs these veins.
Academic Accuracy: ☐1 ☐2 ☐3 x4 ☐5
Empathy Score: ☐1 ☐2 ☐3 x4 ☐5

C) The treated veins close and are gradually absorbed by the body or shrink.
Academic Accuracy: ☐1 ☐2 ☐3 x4 ☐5
Empathy Score: ☐1 ☐2 x3 ☐4 ☐5

D) The embolized veins close and blood flow is redirected to healthy veins. This reduces pressure in the testicle and improves symptoms.
Academic Accuracy: ☐1 ☐2 ☐3 ☐4 x5
Empathy Score: ☐1 ☐2 ☐3 x4 ☐5
