# Supplementary figures and images for: Comparison of artificial intelligence models and physicians in patient education for varicocele embolization: a double-blind randomized controlled trial
Source: Front Radiol. 2025 Oct 14;5:1682725. doi: 10.3389/fradi.2025.1682725 (PMC12558931; doi:10.3389/fradi.2025.1682725)

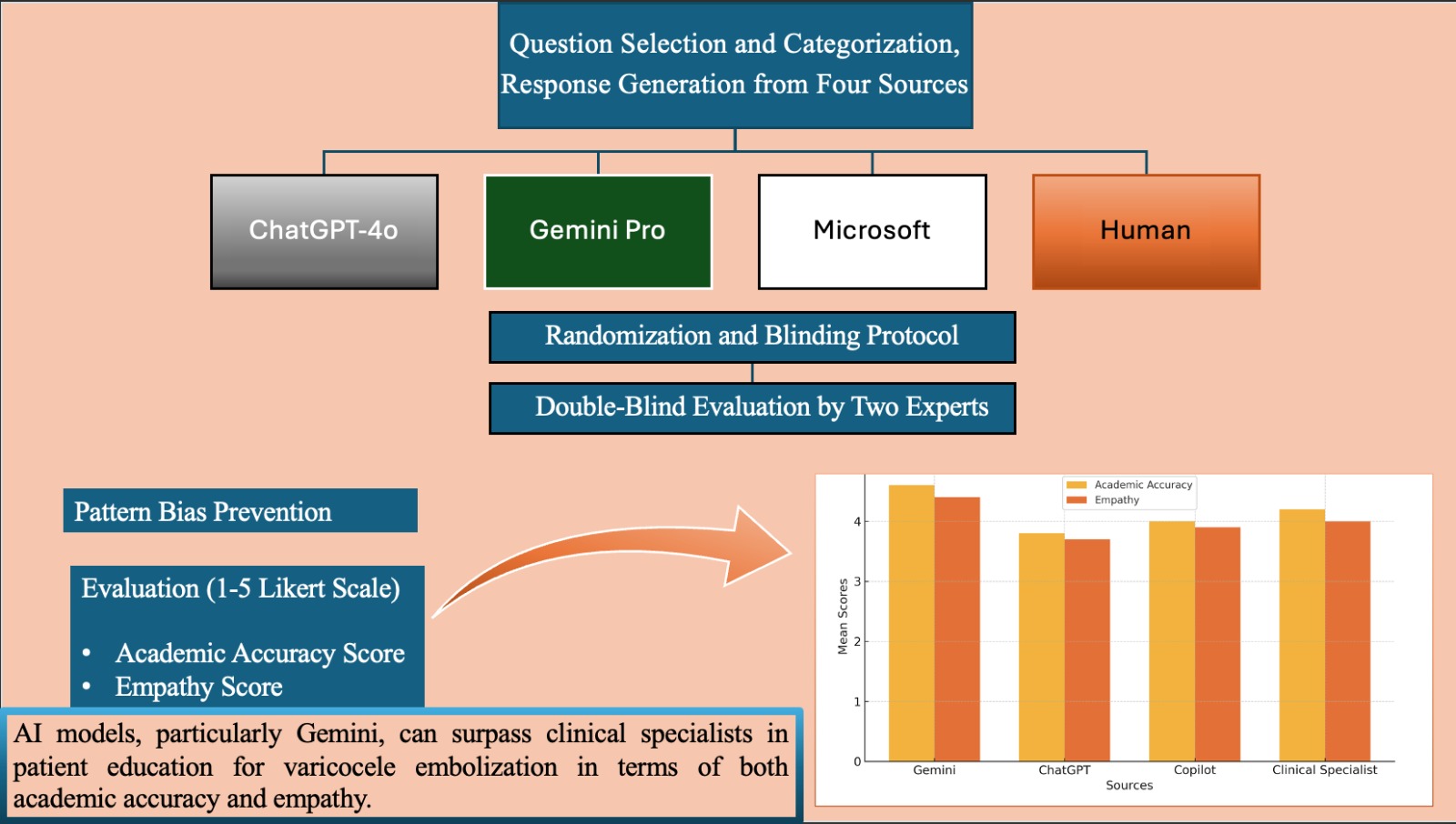

Supplement: Supplementary file 6 [file Image1.jpeg]
